# A taxonomic review of *Boreus* (Mecoptera, Boreidae) with descriptions of two new Alaskan species

**DOI:** 10.3897/zookeys.1267.170946

**Published:** 2026-01-23

**Authors:** Taylor L. Kane, Derek S. Sikes, Nathan Schiff

**Affiliations:** 1 University of Alaska Museum, Fairbanks, AK, USA University of Alaska Fairbanks Fairbanks United States of America; 2 Department of Biology & Wildlife, University of Alaska Fairbanks, Fairbanks, AK, USA University of Alaska Museum Fairbanks United States of America

**Keywords:** DNA barcoding, molecular phylogeny, morphology, snow scorpionflies, species delimitation

## Abstract

*Boreus* (Mecoptera, Boreidae) species are reviewed. Two new Alaskan species are described (*Boreus tananaensis* Kane, **sp. nov**., *Boreus timaryi* Kane, **sp. nov**.), a previously synonymized species is resurrected (*Boreus gracilis* Carpenter, 1935, **stat. res**.), and morphological descriptions are provided for all five Alaskan species. A key to male and female Alaskan *Boreus* species is provided. An estimate of the mitochondrial gene tree, based on COI DNA barcodes, is used to infer relationships, and morphological species are tested using five molecular species delimitation methods. What is known about the subgeneric classification of *Boreus*, and how Alaskan species are classified, is discussed. A checklist of all 33 currently valid species of the family Boreidae is provided.

## Introduction

Boreidae is a family in the order Mecoptera, composed of small, brachypterous insects that are poorly understood and rarely collected. With 33 valid species, Boreidae is the third most species-rich family in the order Mecoptera. The family Boreidae consists of two subfamilies and three extant genera: Boreinae (*Boreus*, *Hesperoboreus*) and Caurininae (*Caurinus*). *Boreus* is the most species-rich genus in the family with 29 valid species compared to two *Hesperoboreus* species and two *Caurinus* species. While *Caurinus* and *Hesperoboreus* have ranges restricted to the North American Pacific West, *Boreus* has a Holarctic distribution.

Like all other Boreidae, *Boreus* are brachypterous. Males have long, claw-like wings used to clasp the female during copulation, while female wings are nearly absent, reduced to small, oval-shaped sclerotized pads. Boreidae also differ from other Mecopterans in their ability to jump. When disturbed, *Boreus* will jump several times, sometimes up to 12 inches (Penny 1977), before falling to their side and lying prone. *Boreus* have also been observed jumping repeatedly in the same direction on top of the snow, apparently as a mode of dispersal (Shorthouse 1979; Burrows 2011; Hågvar and Østbye 2011).

As their common names “snow scorpionflies” or “snow fleas” suggest, *Boreus* are cold-adapted, and often winter active. *Boreus* appear to generally have a two-year life cycle (Beutel et al. 2019), although the phenology of most species is poorly known. Most species are active as adults between November and April (Penny 1977; Beutel et al. 2019) and are often collected on top of the snow on days ranging between -5 to +5 °C (Cockle 1917; Hågvar and Østbye 2011; Hendricks 2025). In contrast, adult *Boreus* in Alaska have only been collected in the spring, summer, and fall, between April and September. How Alaskan species overwinter, and their general phenology, is unknown.

*Boreus* can be found in a wide range of macrohabitats, from beaches near sea level to high altitude mountains and across a wide latitudinal gradient, in both forested areas and open grasslands (Penny 1977; Beutel et al. 2019; Hendricks 2025). *Boreus* live in and feed on mosses to some degree during all their life stages (Beutel et al. 2019), although it has been suggested that adult *Boreus* may also opportunistically feed on dead insects (Withycombe 1922).

The factors that influence the distribution of *Boreus* are not well understood. Even in widespread species such as *Boreus hyemalis* (Linnaeus, 1767), *Boreus* exhibit uneven, clumped distributions (Pyszko et al. 2024), and many species have ranges restricted to small areas. These factors, along with their unusual phenology make *Boreus* difficult to collect. Because of this, *Boreus* are usually collected by hand through opportunistic sampling, either on top of snow or from moss, although passive sampling via pitfall traps has been found to be effective in some cases (Blades 2002; Hågvar and Østbye 2011; Looney et al. 2019). However, pitfall samples often become engorged, which can distort characters from their in situ appearances, particularly body length (Penny 1977; Blades 2002).

The majority of the 29 currently valid *Boreus* species, including all three previously named Alaskan species, were described in the 19^th^ and 20^th^ centuries. Prior to this publication, only two species of *Boreus* were described in the 21^st^ century – *Boreus insulanus* Blades, 2002 from Vancouver Island and *Boreus altaicus* Nikolajev, 2015 from Kazakhstan. Three different methods to classify *Boreus* species have been proposed. Lestage (1940) proposed splitting the genus into two genera. Penny (1977) suggested a classification consisting of four species groups and four species subgroups, and Nikolajev (2003) named three subgenera based on his study of Palearctic species. These proposed classifications are non-concordant and based only on morphological data.

Palearctic *Boreus* have received recent attention, with studies documenting significant range expansions for Palearctic *Boreus* species (Stolbov et al. 2018; Vertyankin and Zaycev 2021; Wang and Jia 2024) and examining the relationships between the European *Boreus* species (Kaspřák 2012; Zangl et al. 2021; Strohmeier 2023). However, limited work has been done on Nearctic *Boreus* in the last quarter century, and a genus-level molecular phylogeny of *Boreus* species has never been estimated.

### Summary of Alaskan *Boreus*

**Figure 1.**
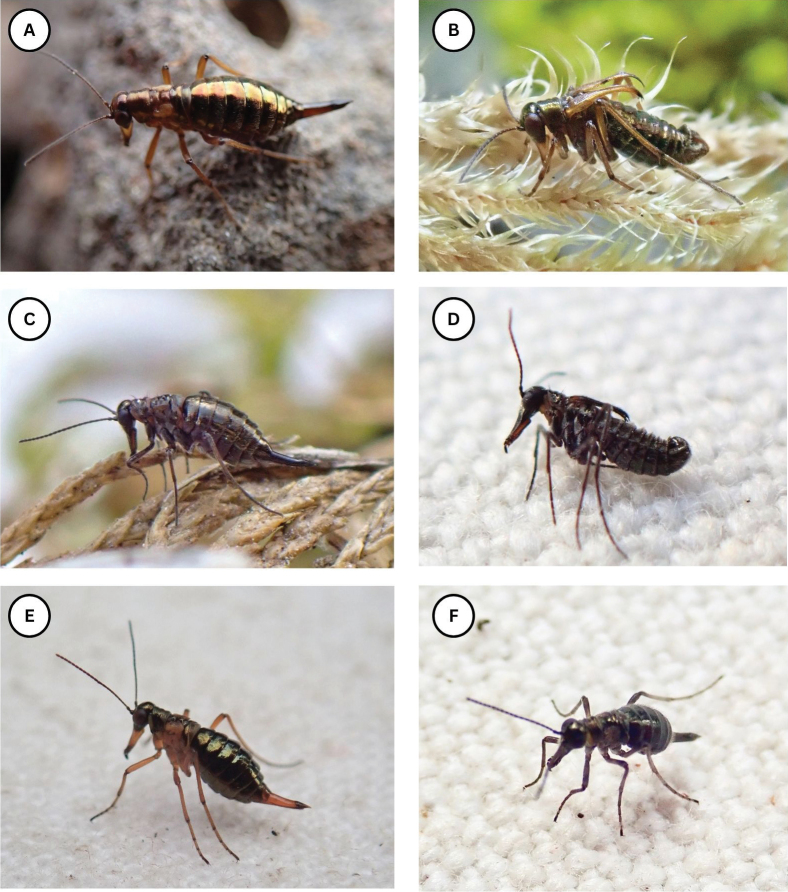
Photos of living Alaskan *Boreus* spp. **A**. Female *Boreus borealis*; **B**. Male *B. borealis*; **C**. Female *B. tananaensis* sp. nov.; **D**. Male *B. tananaensis* sp. nov.; **E**. Female *B. intermedius*; **F**. Female *B. gracilis*. Photo credits: **A**: DSS, all others: TLK.

**Figure 2.**
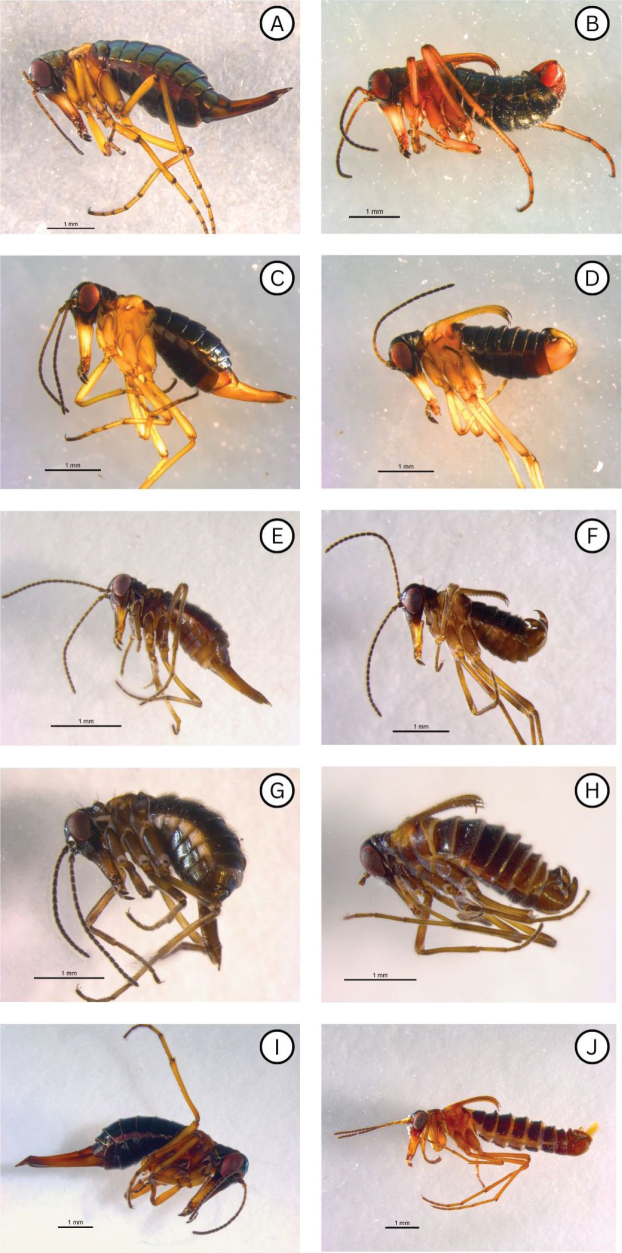
Alaskan *Boreus* species: *Boreus borealis*; **A**. Female (UAM:Ento:235218); **B**. Male (UAM:Ento:235219); *Boreus intermedius*; **C**. Female (UAM:Ento:451067); **D**. Male (UAM:Ento:477812); *Boreus tananaensis* sp. nov.; **E**. Female (UAM:Ento:504865); **F**. Male (UAM:Ento:504866); *Boreus gracilis*; **G**. Female (UAM:Ento:457836); **H**. Male (UAM:Ento:477811); *Boreus timaryi* sp. nov.; **I**. Female (UAM:Ento:406584); **J**. Male (UAM:Ento:507961). Photo credits: TLK.

**Figure 3.**
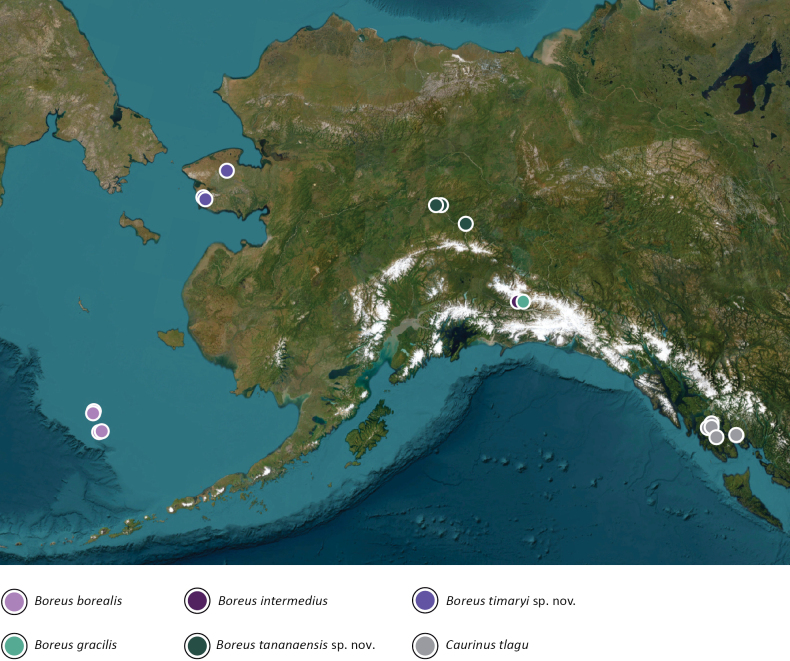
Known records of Alaskan Boreidae species.

The first documented Alaskan *Boreus* was *Boreus borealis* Banks, 1923 (Figs 1A, 1B, 2A, 2B), from St. Paul Island, within the Pribilof Islands (Fig. 3). *Boreus borealis* was described from four specimens (2 ♂♂, 2 ♀♀), collected in 1914 by Alvin G. and Elsie G. Whitney (McAtee et al. 1923). Two additional *B. borealis* specimens were collected from the same locality in 1925 by A. Christofferson, who worked as an engineer on St. Paul Island (Penny 1977; Lindsay and Lindsay 2010). In 1975 Michael Torrey, the son of a visiting doctor, collected a single male specimen from St. Paul Island. In 2012, four *Boreus* specimens presumably representing *B. borealis* were collected by DSS from St. George Island, the second principal island within the Pribilof Islands, approximately 80 km from the type locality on St. Paul Island.

*Boreus intermedius* Lloyd, 1934 (Figs 1E, 2C, 2D) was described from two specimens (1 ♂, 1 ♀) collected in 1934 in the McCarthy-Kennicott area (Fig. 3), in what would later become a part of Wrangell St. Elias National Park and Preserve. In 1935, four additional specimens were collected from the type locality and nearby areas (Carpenter 1936). Both *B. borealis* and *B. intermedius* have only been collected in Alaska and are presumed endemic to the state.

A second species of *Boreus* (Figs 1F, 2G, 2H) was collected in sympatry with *B. intermedius* in both 1934 and 1935 (Fig. 3). These specimens were originally identified as *Boreus unicolor* Hine, 1901 (Lloyd 1934) a species described from Montana. The specimens were later described as a new species endemic to Alaska, *Boreus gracilis* Carpenter, 1935. During a systematic study of the family Boreidae, it was concluded that *B. gracilis* was a junior synonym of *Boreus nix* Carpenter, 1935, a species present throughout northwestern North America, and the two names were synonymized (Penny 1977).

In April 2016, a single individual representing a putative new *Boreus* species (Figs 1C, 1D, 2E, 2F) was collected by DSS in interior Alaska. In 2018, multiple specimens representing this new species were collected by Takeyuki Nakamura at Quartz Lake, ca 110 km from the first locality (Fig. 3). In July 2016, a second putative new *Boreus* species was found on the Seward Peninsula in western Alaska (Fig. 3). Three specimens of this species were collected during a US National Park Service Bioblitz event held in the Bering Land Bridge National Park and Preserve. Subsequent review of pitfall trap samples from 2012–2013 on the Seward Peninsula yielded ten additional specimens of this putative new species (Sikes et al. 2017). In 2013 another boreid species from the sister subfamily Caurininae, *Caurinus tlagu* Sikes & Stockbridge, 2013, was described from southeast Alaska. The only other species in this subfamily and genus, *C. dectes* Russell, 1979 is known from Washington, Oregon, and potentially British Columbia (iNaturalist 2025).

The existing morphological descriptions of Alaskan *Boreus* are based on a small number of specimens, many of which are damaged and desiccated from being pinned—a practice that is no longer the standard for Boreidae as it results in the loss of multiple descriptive characters. The original type specimens for Alaskan species consisted of small series, many of which have since been lost to science. Because of this, the original descriptions of Alaskan *Boreus* are inadequate and examination of type specimens is not possible for all species. Additionally, no key exists to identify female specimens for any of the Alaskan *Boreus* species. Using existing keys, female specimens can be identified to species group morphologically, but specific identifications are based on locality and associated male specimens (Penny 1977). Because *Boreus* are so rarely collected, and often only collected as singletons, the lack of diagnostic characters for female specimens is particularly problematic, as it effectively results in only 50% of individuals being identifiable using morphological methods. Additionally, the relationship between the Alaskan *Boreus* species and other species is unclear, particularly for the new species from the Seward Peninsula.

Herein we provide a taxonomic review of the genus *Boreus*, including its subgeneric classification, using morphological and genetic data and describe two new species that are presumed endemic to Alaska. We update species descriptions for named Alaskan species, return one invalid species to validity, provide the first morphological key to Alaskan *Boreus*, provide the first genus level molecular phylogeny, and provide a checklist of world Boreidae species.

## Materials and methods

### Collecting

Specimens were obtained primarily via hand collection methods, although some were obtained via pitfall traps. We determined that moss-brushing, a method developed by Loren Russell and Wes Bicha (Russell 2017), was the method best suited to our sampling approach. Moss, typically on vertical surfaces, is disturbed by hand using a gentle back and forth brushing motion over a beating sheet. *Boreus* specimens can be removed from the beating sheet using forceps. This method allowed us to sample a wide range of localities in a short period of time. This sampling method is minimally invasive and non-destructive to habitat, unlike Berlese or Winkler funnel extractors which require uprooting host plants. Additionally, this method allowed us to collect individuals that were in the moss but not active, including pupae.

### Collections

We examined specimens from the following institutions:

**CAS** California Academy of Sciences, San Francisco, California, USA

**MCZ** Museum of Comparative Zoology at Harvard, Cambridge, Massachusetts, USA

**NHRS** Naturhistoriska riksmuseet, Stockholm, Sweden

**RBCM** Royal British Columbia Museum, Victoria, British Columbia, Canada

**SEMC** University of Kansas Biodiversity Institute, Lawrence, Kansas, USA

**UAM** University of Alaska Museum Insect Collection, University of Alaska Fairbanks, Fairbanks, Alaska, USA

### Morphology

We examined specimens from all named North American species including invalid species, and two eastern Asian species (see Suppl. material 2: table SS1 for details on specimens included in the morphological study). We were only able to access physical specimens for a single Russian species, *Boreus semenovi* Pliginsky, 1930. Photographs of the type specimen of the Russian species *Boreus sjoestedti* Navás, 1925 from the Kamchatka Peninsula were supplied by the entomology collection of NHRS. For two eastern Russian species, *Boreus jacutensis* Plutenko, 1984 and *Boreus orientalis* Martynova, 1954, Alaskan specimens were compared to morphological descriptions only.

We examined specimens using a Leica MZ16 microscope and captured character images using a Leica Microsystems DFC425 camera on the same microscope, using LAS (v. 4.13) software for focus stacking. Photographed characters were measured in ImageJ (Schneider et al. 2012). Characters were illustrated in Procreate (v. 5.3.15). We compared characters to species descriptions and type specimens, when available. Species level diagnostic characters for both sexes were identified for all Alaskan species and were used to build dichotomous keys for both males and females of Alaskan species.

Holotypes for the new Alaskan *Boreus* species have been deposited in the UAM. Paratypes for both species have been deposited in the UAM, the CAS, and the MCZ and will be deposited in the RBCM. Specimen data for all types and UAM specimens can be accessed via the online database Arctos (http://arctos.database.museum/).

**Figure 4.**
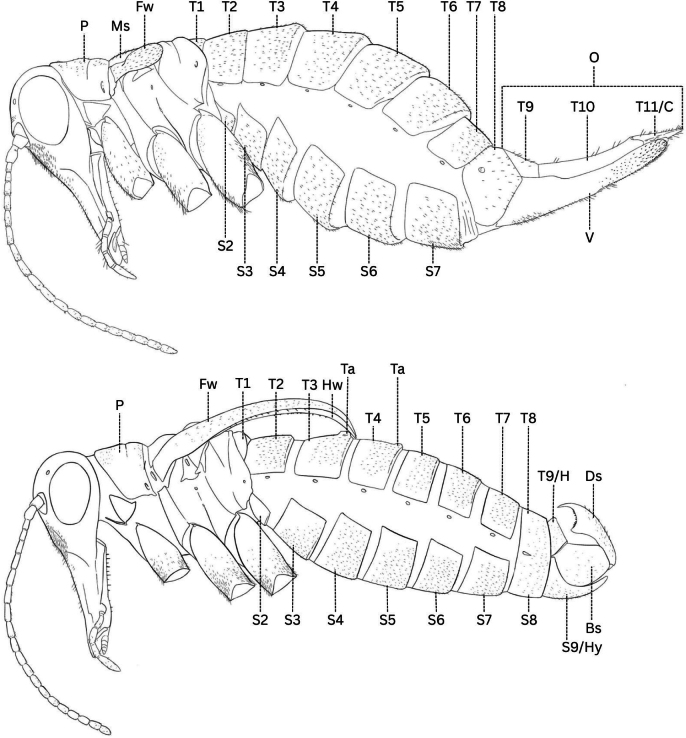
Anatomical guide to *Boreus* female (top) and male (bottom). Abbreviations: Bs – basistyle, C – cerci, Ds – dististyle, Fw – forewing, H – hood, Hw – hindwing, Hy – hypandrium, Ms – mesoscutellum, O – ovipositor, P – pronotum, T1–11 – tergum 1–11, Ta – tergal apophysis, S2–S9 – sternum 2–9, V – valvula.

Both discrete and continuous characters were measured, including:

**Overall**: body length (Fig. 4, Suppl. material 1: fig. S1A, B), coloration, pilosity.

**Figure 5.**
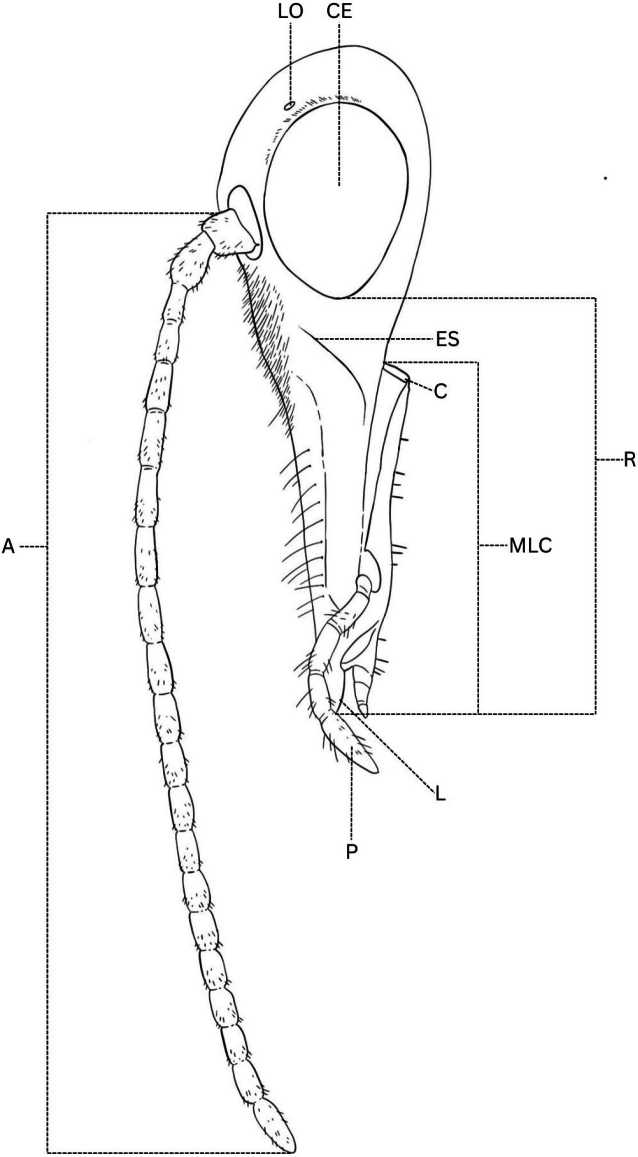
*Boreus* head anatomy. Abbreviations: A – antenna, C – cardo, CE – compound eye, ES – epistomal suture, L – labrum, LO – lateral ocellus, MLC – maxillolabial complex, P – palps, R – rostrum.

**Head**: head length, rostrum length, maxillolabial complex (MLC) length (Fig. 5, Suppl. material 1: fig. S2A–C), ratio of MLC length to rostrum length, description/number/placement of hairs and spines on head.

**Eyes**: eye length (Suppl. material 1: fig. S2D), ratio of eye to rostrum length, color of eyes, presence and size of median ocellus, size and placement of lateral ocelli.

**Antennae**: Number of antennomeres, relative length of antennomeres, coloration of antennae.

**Thorax**: Thorax color, number of spines on anterior margin of pronotum, number of spines on posterior margin of pronotum, size of spines, presence and number of spines on mesoscutellum.

**Female wings**: forewing length (Suppl. material 1: fig. S3A), visibility of hindwings, color.

**Male wings**: forewing length (Suppl. material 1: fig. S3G), number of inner forewing spines, number of outer forewing spines, relative length of terminal forewing spine, number of hind wing spines, presence of hairy pad on hind wings.

**Female terminalia**: Length of ovipositor (Suppl. material 1: fig. S3B), length of valvulae (Suppl. material 1: fig. S3C), length of segment 9 (Suppl. material 1: fig. S3D), length of segment 10 (Suppl. material 1: fig. S3E), length of segment 11 (Suppl. material 1: fig. S3F), ratio of ovipositor to rostrum length, ratio of cerci to ovipositor length, ovipositor color.

**Figure 6.**
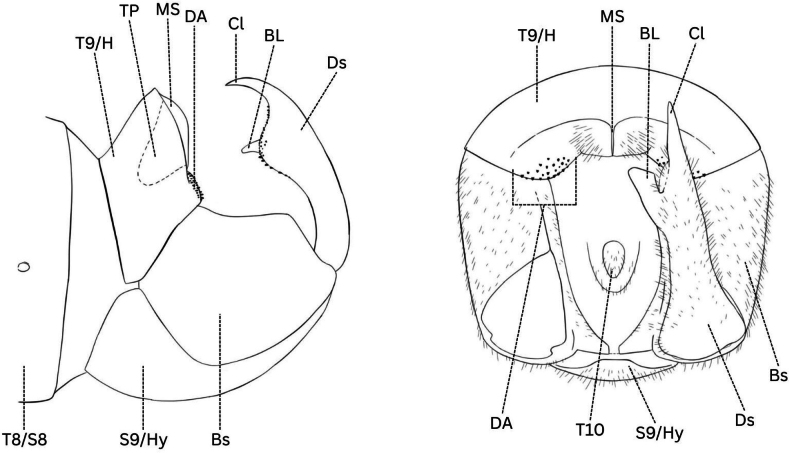
*Boreus* male terminalia anatomy viewed laterally (left) and dorsally (right). Abbreviations: BL – basal lobe, Bs – basistyle, Cl – claw, DA – denticular area, Ds – dististyle, H – hood, Hy – hypandrium, MS – median septum, S8–9 – sternum 8–9, TP – tergal pockets, T8–10 – tergum 8–10.

**Male terminalia**: Fusion of 8^th^ tergum and sternum, fusion of 9^th^ tergum and sternum, hypandrium length, shape of distal edge of hypandrium, size/shape of 9^th^ tergum (hood), size/shape of hood septum, number of hood denticles, shape of dististylar basal lobe, description of hairs on dististyles, number and placement of denticles on dististyles (Fig. 6).

### Molecular methods

University of Alaska Museum specimens were processed at the Canadian Centre for DNA Barcoding (CCDB). A single leg was removed from each specimen for minimally destructive sampling, and DNA was extracted, amplified, and sequenced at the CCDB. These sequences, as well as additional COI sequences provided by NS that were sequenced at the USDA Forest Service, Southern Research Station, Center for Bottomland Hardwoods Research, in Stoneville Mississippi, were archived with the Barcode of Life Data Systems (BOLD). Additional *Boreus* COI sequences used were accessed through BOLD’s public repository. Sequences were manually examined, and any suspected of misidentification or contamination were removed from the data set. From the 69 public BOLD COI sequences for *Boreus westwoodii* Hagen, 1866, ten sequences were randomly subsampled to be included in analyses. In total, 17 *Boreus* species were included in the molecular analyses, including 14 of the 15 known Nearctic species and three of the 14 known Palearctic species. After pruning, 125 *Boreus* sequences, three *Hesperoboreus brevicaudus* sequences, and one *Caurinus dectes* sequence were included in the final analyses (see Suppl. material 2: table SS1 for details on specimens included in the molecular datasets).

All analyses were conducted on the full dataset (hereafter the ‘All *Boreus* dataset’) as well as a subsampled dataset containing only the Alaskan *Boreus* sequences with one *Hesperoboreus brevicaudus* sequence as the outgroup (hereafter the ‘AK *Boreus* dataset’). We inferred COI barcode trees using maximum-likelihood in IQ-TREE (Minh et al. 2020) with model selection based on Bayesian Information Criterion scores using IQ-TREE’s built in ModelFinder (Kalyaanamoorthy et al. 2017). Branch support was reported as ultrafast bootstrap (UFBS) (Minh et al. 2013) and Shimodaira–Hasegawa-like approximate likelihood-ratio test (SH-aLRT) (Guindon et al. 2010) values. Branch support was only reported for branches above species level, unless species were non-monophyletic. In the All *Boreus* dataset tree, branches above the species level with UFBS support < 80% were collapsed using iTOL v. 7.2 (Letunic and Bork 2021). Branches were considered well supported if they had both UFBS ≥ 95% and SH-aLRT ≥ 80%. Branches were considered moderately supported if they had either UFBS ≥ 95% or SH-aLRT ≥ 80%. Branches were considered weakly supported if they had both UFBS < 95% and SH-aLRT < 80%.

### Species delimitation methods

We tested our morphological species delimitation hypotheses against five molecular species delimitation methods: two tree-based algorithms – a generalized mixed Yule coalescent method (GMYC; Fujisawa and Barraclough 2013) and a multi-rate Poisson tree processes method (mPTP; Kapli et al. 2017); two distance based algorithms – the assemble species by automatic partitioning model (ASAP; Puillandre et al. 2021) and the automatic barcode gap discovery method (ABGD; Puillandre et al. 2012); and one clustering algorithm – BOLD’s barcode index number system (BIN; Ratnasingham and Hebert 2013). For the GMYC analyses an ultrametric tree was created from each dataset in BEAST (Bouckaert et al. 2019) based on a Yule model. The MCMC length was initially set to 10,000,000 for each tree and increased as needed until all parameters had an estimated sample size greater than 200. GMYC species delimitation was conducted in R using the ‘splits’ package (Ezard et al. 2009). The mPTP analyses were based on the IQ-TREE generated maximum likelihood trees and used the generated FASTA alignments to estimate minimum branch lengths and were conducted on the online MCMC-mPTP webservice (https://mcmc-mptp.h-its.org/). The ASAP and ABGD analyses were run on Spart Explorer (https://spartexplorer.mnhn.fr/) and were based on a simple distance substitution model.

**Figure 7.**
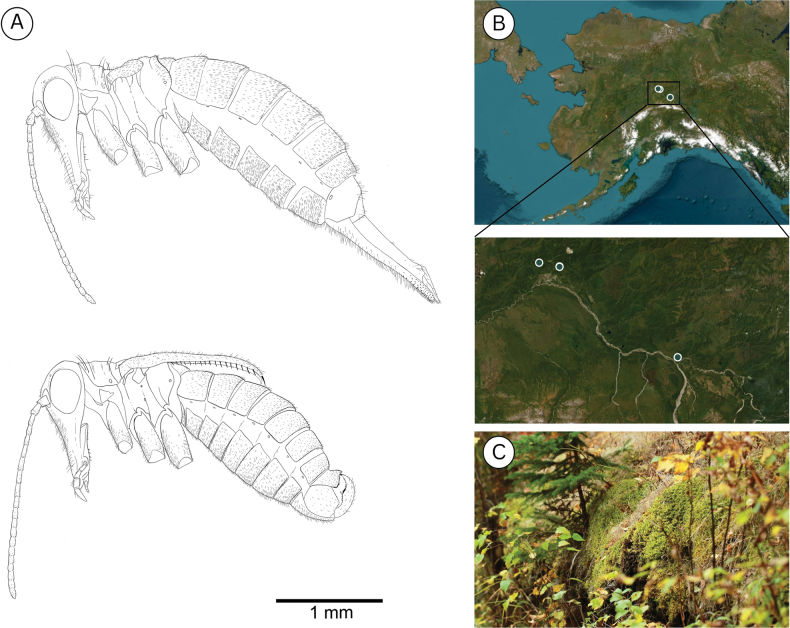
**A**. *Boreus tananaensis* sp. nov. female (top) and male (bottom) viewed laterally. **B**. Collection localities for *B. tananaensis*. **C**. Habitat of *B. tananaensis*. Photo credit: Takeyuki Nakamura.

For each Alaskan species, distribution maps were generated in ArcGIS Pro, based on historic and modern collection events. Habitat photos (Figs 7C, 10C, 11C, 12C) were taken using an Apple iPhone or OLYMPUS Tough TG-4 digital camera, and live specimen photos (Fig. 1) were taken with an OLYMPUS Tough TG-4 or TG-6 digital camera.

**Figure 8.**
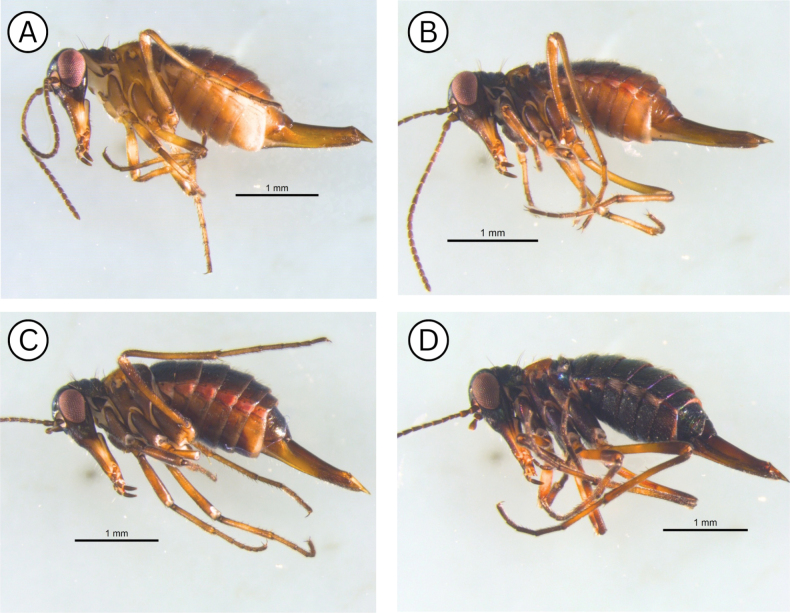
Coloration difference in *Boreus tananaensis* specimens collected in the fall and spring: **A**. September 9, 2023 (UAM:Ento:492024); **B**. September 12, 2024 (UAM:Ento:504865); **C**. September 12, 2024 (UAM:Ento:504856); **D**. April 27, 2025 (UAM:Ento:508002). Photo credits: TLK.

**Figure 9.**
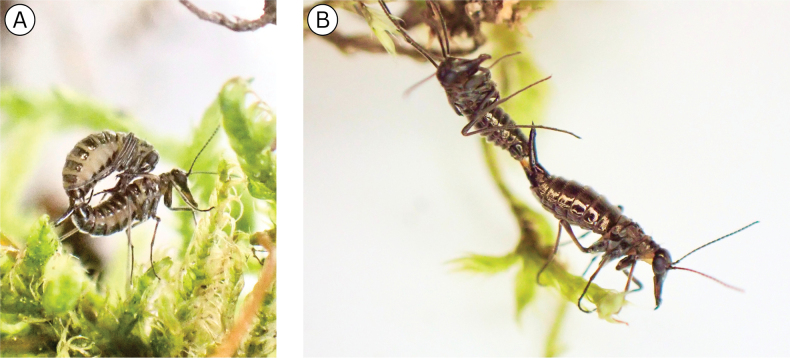
*Boreus tananaensis* mating. **A**. 2^nd^ phase of copulation (photographed 16 September 2022); **B**. 3^rd^ phase of copulation (photographed 27 April 2025) as described by Lestage (1941). Photo credits: TLK.

**Figure 10.**
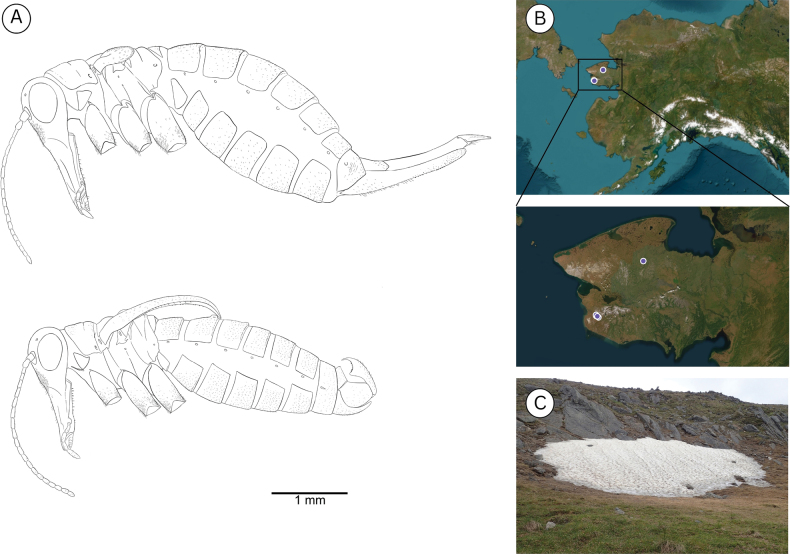
**A**. *Boreus timaryi* sp. nov. female (top) and male (bottom) viewed laterally. **B**. Collection localities for *B. timaryi*. **C**. Habitat of *B. timaryi* holotype. Photo credit: DSS.

**Figure 11.**
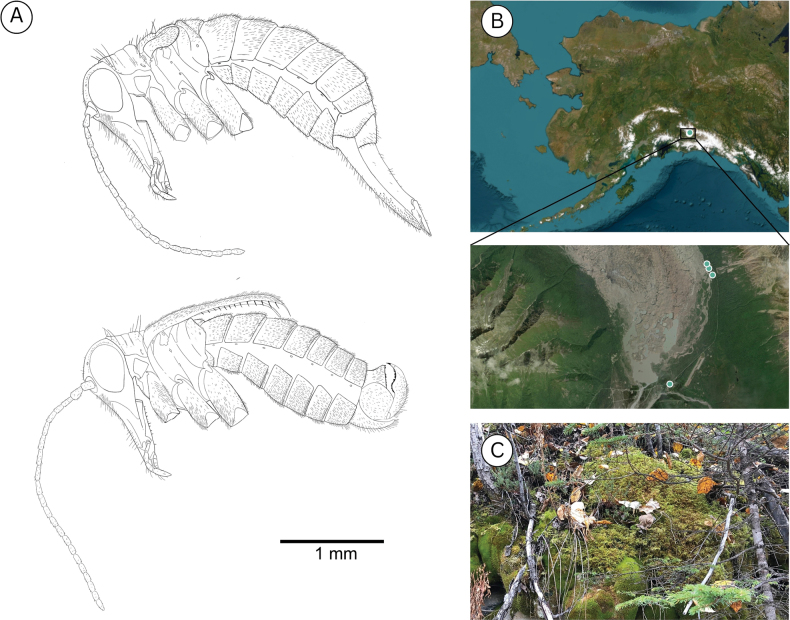
**A**. *Boreus gracilis* female (top) and male (bottom) viewed laterally. **B**. Collection localities for *B. gracilis*. **C**. Habitat of *B. gracilis*. Photo credit: TLK.

**Figure 12.**
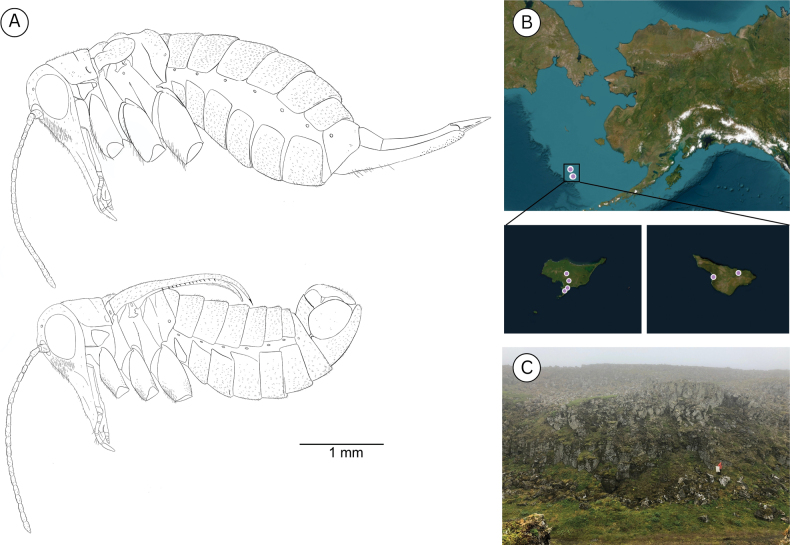
**A**. *Boreus borealis* female (top) and male (bottom) viewed laterally. **B**. Collection localities for *B. borealis*. **C**. Habitat of *Boreus borealis* on St. Paul Island. Photo credit: TLK.

## Results

### Systematics

#### Boreus tananaensis

Taxon classificationAnimaliaMecopteraBoreidae

Kane sp. nov.

77B7FFFA-9882-5E1C-951F-17D3941E12A3

https://zoobank.org/9F01B5B9-6387-4DF3-B987-6F0AE8A6DCF4

Figs 1C, 1D, 2E, 2F, 7, 8, 9, 19C, 20B, 21B, 22B, 26B

##### Type material.

Description based on 29 ♂♂ in ethanol, 1 ♂ pinned, 23 ♀♀ in ethanol, and 1 ♀ pinned: ***Holotype*** (UAM). United States – **Alaska** • ♀; Quartz Lake Recreation Area; 64.199658, -145.828804; 12 Sept. 2020; DSS and TLK leg.; UAM:Ento:442469. ***Paratypes*** (17 ♂♂, 22 ♀♀ – 33 UAM, 2 CAS, 2 MCZ, 2 RBCM). United States – **Alaska** • 1 ♂; Fairbanks, Fitz Ct.; 64.90114, -147.52807; 10 Apr. 2016; DSS leg.; UAM:Ento:334169. • 1 ♀; Same collection data as previous; 64.899906, -147.526471; 16 Sept. 2022; UAM:Ento:476981. • 1 ♀; Fairbanks, Goldstream Rd.; 64.93915, -147.84528; 14 Sept. 2020; TLK leg.; UAM:Ento:442474. • 1 ♂, 1 ♀; Quartz Lake State Recreation Area; 64.199658, -145.828804; 15 Sept. 2018; Takeyuki Nakamura leg.; UAM:Ento:378804, UAM:Ento:378805. • 4 ♂♂, 3 ♀♀; Same collection data as type specimen; 12 Sept. 2020, DSS and TLK leg.; UAM:Ento:442467, UAM:Ento:442468, UAM:Ento:442472, UAM:Ento:442473, UAM:Ento:442487, UAM:Ento:442505, UAM:Ento:442506. • 1 ♀; Same collection data as previous; 2 May 2022; TLK leg.; UAM:Ento:471748. • 1 ♀; Same collection data as previous; 15 May 2022; UAM:Ento:477847. • 2 ♂♂, 1 ♀; Same collection data as previous; 25 Sept. 2022; UAM:Ento:477813, UAM:Ento:477818, UAM:Ento:477819. • 1 ♂; Same collection data as previous; 11 May 2023; UAM:Ento:483367. • 1 ♂, 3 ♀♀; Same collection data as previous; 9 Sept. 2023; UAM:Ento:492015, UAM:Ento:492023, UAM:Ento:492024, UAM:Ento:492026. • 5 ♂♂, 6 ♀♀; Same collection data as previous; 12 Sept. 2024; UAM:Ento:504855, UAM:Ento:504856, UAM:Ento:504857, UAM:Ento:504860, UAM:Ento:504862, UAM:Ento:504863, UAM:Ento:504864, UAM:Ento:504865, UAM:Ento:504866, UAM:Ento:504867, UAM:Ento:506621. • 3 ♂♂, 3 ♀♀; Same collection data as previous; 27 Apr. 2025; UAM:Ento:508001, UAM:Ento:508002, UAM:Ento:508003, UAM:Ento:508004, UAM:Ento:508005, UAM:Ento:508006.

##### Other material.

(13 ♂♂, 1 ♀) United States – **Alaska** • 1 ♂; Quartz Lake State Recreation Area; 12 Sept. 2020; DSS and TLK leg.; UAM:Ento:442471. • 4 ♂♂; Same collection data as previous; 9 Sept. 2023; UAM:Ento:495423, UAM:Ento:495424, UAM:Ento:492025, UAM:Ento:492027. • 6 ♂♂; Same collection data as previous; 25 Sept. 2023; UAM:Ento:492080, UAM:Ento:492134, UAM:Ento:492135, UAM:Ento:492136, UAM:Ento:492137, UAM:Ento:492138. • 2 ♂♂, 1 ♀,: Same collection data as previous; 12 Sept. 2024; UAM:Ento:504858, UAM:Ento:504859, UAM:Ento:504861.

##### Diagnosis.

*Boreus tananaensis* can be differentiated from non-Alaskan members of the *brumalis* subgroup of the *nivoriundus* species group sensu Penny 1977 (*Boreus brumalis* Fitch, 1847, *Boreus bomari* Byers & Shaw, 1999, *Boreus pilosus* Carpenter, 1935, *B. insulanus*, and *B. nix*) by the greatly reduced or absent median ocellus, and from most members of the species group by the presence and relative length of cruciate bristles on the female mesonotum, which are similar in length to pronotal bristles and distinctly longer than the surrounding hairs. Both sexes of *Boreus tananaensis* differ from those of its apparent sister species (see below) *B. gracilis* by the length of the thoracic and abdominal hairs, which are shorter than half the length of the thoracic bristles in *B. tananaensis* and exceed half the length of the thoracic bristles in *B. gracilis*. *Boreus tananaensis* can also be differentiated from *B. gracilis* by coloration; *B. tananaensis* is brown with a green-blue iridescence, while *B. gracilis* is brown with a gold iridescence. This difference in coloration is less obvious in pinned specimens. Male *B. tananaensis* also differ from *B. gracilis* in the shape of the cleft of the tergal hood, which is short and triangular as opposed to long and narrow in *B. gracilis*, and the posterior margin of the denticular area, which curves in towards the cleft in *B. tananaensis* and is straight in *B. gracilis*.

##### Description.

***Head***. Head dark with green-blue iridescent sheen from top of head to part way down rostrum. Maxillolabial complex non-iridescent, brown fading to yellow distally. Head length measured from top of head to distal tip of labrum 1.06–1.53 mm (female mean = 1.33, male mean = 1.25). Eyes reddish-purple. Diameter of eye measured parallel to direction of head 0.34–0.52 mm (female mean = 0.43, male mean = 0.43). Lateral ocelli about diameter of ocellus away from compound eye margin. Median ocellus reduced, significantly smaller than lateral ocelli, or absent. Epistomal suture not reaching the margin of the maxillolabial complex. Antennae with 20–23 antennomeres (female mode = 20, male mode = 21). Antennae dark brown, darker distally. Fine white hairs covering entire head, most dense and long on frontal region. Maxillolabial complex to rostrum length ratio 0.76–0.90 (female mean = 0.81, male mean = 0.84).

***Thorax***. Pronotum same color as top of head and abdominal terga, with two transverse furrows. Anterior edge of pronotum with 2–4 (mode = 2) bristles on each side. Posterior edges of pronotum with 2–3 (mode = 2) bristles on each side. Mesonotum similar in color to pronotum. Mesoscutellum with two cruciate bristles similar in length to pronotum bristles in females, no bristles in males. Metanotum similar in color to mesonotum, without bristles.

***Legs***. Leg segments brown, darker adjacent to joints, giving a striped appearance. Coxae covered in long, fine, white hairs; densest on anterior face. All other leg segments covered in short, fine, white hairs. Femur with apical-femoral spine present, long, pointed, and black, without additional spines. Tibia with two long, translucent-brown apical spurs. Tibia and tarsi with two rows of yellow spines on ventral surface, no spines on dorsal surface.

***Female wings***. Forewings yellow-brown, translucent, covered in short, sparse, fine, dark hairs, reduced to small ovals 0.25–0.40 mm (mean = 0.33) in length, covering entire hindwing.

***Male wings***. Forewings yellow-brown, similar in color to legs, darker distally. Forewings covered in medium-length, dense, fine, dark hairs. Forewing length 1.08–1.56 mm (mean = 1.31). Outer margin of forewing tapers evenly when viewed dorsally. Margins of forewings bear 12–21 (mode = 13) inner spines and 11–22 (mode = 16) outer spines. Terminal spine of forewing significantly longer than penultimate inner spine. Hindwings approximately same length as forewings, ending in flattened, curled tips. Hindwings with 7–11 (mode = 11) peg-like spines which decrease in size distally. Distal half of hindwings with pad of dense, short white hairs, ending before curved tip.

***Female abdomen***. Terga and sterna brown with blue-green iridescent sheen, covered in long, fine, dense, near-translucent hairs. Intersegmental membrane varies in color from red to pale yellow. Tergum 8 with fine wrinkles and short, fine, sparse, near-translucent hairs. Female terminalia (segments 9–11) brown, similar in color to legs. Ovipositor (segments 9–11) 1.00–1.33 mm (mean = 1.17) in length. Ovipositor to rostrum length ratio 1.22–1.54 (mean = 1.35). Tergum 9 0.04–0.18 mm (mean = 0.10) in length, with short, fine, sparse, near-translucent hairs. Tergum 10 0.55–0.93 mm (mean = 0.81) in length, with few hairs along dorsal surface. Segment 11 and cerci fused, 0.26–0.38 mm (mean = 0.31) in length, with short, fine, sparse translucent hairs on dorsal and ventral surfaces. Ventral ovipositor valves (valvulae) 0.90–1.17 mm (mean = 1.03) in length, with medium-length, fine, sparse, translucent hairs on ventral surface transitioning to short, peg-like spines with hairs emerging from them on the distal third of the ventral surface.

***Male abdomen***. Terga and sterna 1–8 colored as in female. Tergum 8 and sternum 8 not fused. Tergum 9 and sternum 9 not fused. Tergum 9 with a small hood extending laterally to median of the denticular areas, with short median septum and deep cleft. Cleft triangular, narrowing underneath hood. Tergal pockets not fully separated, covered in fine, short white hairs. Denticular area does not extend into tergal pockets. Sternum 9 (hypandrium) is broadly triangular with notched distal edge, reaching to distal edge of basistyles, covered in long, fine, dense, white hairs. Dististyles brown to yellow, covered in long, fine, dense, white hairs. Basal lobe of dististyle square, separated from base of dististyle by triangular cleft. Outside of dististyle with denticles extending from base of dististylar claw to basal lobe. Tergum 10 and sternum 10 reduced, small oval sclerites, covered in short, fine hairs.

***Body length***. Females: preserved in ethanol 3.05–4.44 mm (mean = 3.83), pinned 3.39 mm. Males: preserved in ethanol 2.19–3.22 mm (mean = 2.68), pinned 1.71 mm.

***Variation***. The shape of distal margin of the hypandrium varies significantly in male *B. tananaensis*. In all specimens, the hypandrium is emarginate, but the width and the depth of the notch vary significantly. As has been documented in other *Boreus* species, *B. tananaensis* shows temporal variation in coloring related to development. The coloration differences between *B. tananaensis* and *B. gracilis* noted above are consistent regardless of the darkness of specimens although the iridescence is most noticeable on darker areas. *Boreus tananaensis* specimens collected in the fall (September) have multiple forms. In the majority of specimens collected the thorax, legs, abdominal sterna, and terminalia are significantly paler than the abdominal terga and head (Fig. 8A). Some specimens exhibit an intermediate form, where the abdominal terga and sterna are the same dark color, but the legs, wings, and terminalia are still significantly paler (Fig. 8B), and some individuals have the same coloration as the spring-collected specimens (Fig. 8C). Specimens collected in the spring (April and May) are dark and have abdominal sterna and terga that are the same color, while their legs, wings, and terminalia are slightly paler than the abdomen (Fig. 8D).

***DNA***. We obtained eight COI sequences for *B. tananaensis*: two from specimens collected in Fairbanks, and six from specimens collected at Quartz Lake. There were 17 variable sites within the sequences. BIN: BOLD:ADR7546. Holotype sequence: UAMIC4046-21.

##### Etymology.

The specific epithet refers to the Tanana Valley in Interior Alaska, where all specimens representing this species were collected. The name Tanana comes from the Athabaskan Tena Dona’, meaning upriver trail (Kari 1985).

##### Distribution.

*Boreus tananaensis* has been collected from three localities in the Tanana Valley of Interior Alaska (Fig. 7B). The two localities in the greater Fairbanks area have yielded only singleton specimens, while the Quartz Lake locality consistently yielded a large number of *B. tananaensis* specimens. It seems likely that *B. tananaensis* is more widespread in Interior Alaska, but other localities have not yet been identified.

##### Ecology.

All specimens were collected from mossy embankments in boreal forest consisting predominantly of mixed spruce (*Picea mariana* and *Picea glauca*), birch (*Betula* sp.), and willows (*Salix* spp.). The population at Quartz Lake was collected on a mossy embankment that consists primarily of a mixture of *Polytrichum strictum* and multiple feather moss species. Specimens were collected in spring (April and May) and fall (September) on sunny, cloudy, and rainy days when the temperature ranged from 4–17 °C (mean = 10 °C). In the spring, specimens were collected while there was still partial snow cover, but *B. tananaensis* was never observed active on the snow, and only ever collected from moss. Even on days when the ambient temperature was relatively high (~10 °C) specimens that were placed on nearby snowbanks became unresponsive. This suggests that *B. tananaensis* may not be active on the snow, as is seen in other *Boreus* species. This could be explained by the significantly colder temperatures observed in Interior Alaska, which regularly drops below -40 °C during the winter.

*Boreus tananaensis* was observed mating on three occasions, 15 September 2018 (by Takeyuki Nakamura), 16 September 2022 (by TLK), and 27 April 2025 (by TLK). On 16 September 2022 the specimens were collected mid-coitus and continued to mate for an additional hour while being observed. On 27 April 2025 two male and three female specimens were collected individually and placed in a vial with moss. One pair began copulating almost immediately and remained joined for at least three hours. Both instances of mating observed by TLK (Fig. 9) were consistent with the mating behavior previously described in *Boreus* (Lestage 1941).

The sex ratios of *B. tananaensis* appear to be approximately equal, with a total of 36 ♂♂ and 38 ♀♀ observed, and similar ratios in both the fall (31 ♂♂ and 33 ♀♀) and spring (5 ♂♂ and 6 ♀♀). At the beginning of sampling, all specimens found were collected. During later collecting trips, to reduce the potential impact on this population, females that appeared gravid were not collected. The number of specimens reported here are the numbers that were observed but not necessarily collected.

#### Boreus timaryi

Taxon classificationAnimaliaMecopteraBoreidae

Kane sp. nov.

9EB93EFD-CA7E-535D-8CB0-085CAED2F58A

https://zoobank.org/DA35C2FA-45EA-4B90-BD1A-C2D7F65221A8

Figs 2I, 2J, 10, 19B, 20A, 23A

##### Type material.

Description based on 7 ♂♂ in ethanol and 6 ♀♀ in ethanol: ***Holotype*** (UAM). United States – **Alaska** • ♀; Seward Peninsula, Bering Land Bridge National Preserve; 65.83713, -164.58995; 9 July 2016; DSS, Kathryn M. Daly, Sam Tocktoo, and Timary Stenek leg.; UAM:Ento:406584. ***Paratypes*** (7 ♂♂, 5 ♀♀, 6 UAM, 1 CAS, 1 MCZ, 1 RBCM), United States – **Alaska** • 1 ♀; Same collection data as type specimen; UAM:Ento:347657. • 1 ♀; Seward Peninsula, Feather River; 64.81, -165.96; 10–16 July 2012; Kelly Overduijn leg.; UAM:Ento:507962. • 1 ♂; Same collection data as previous; 64.82294, -166.01715; 11 July 2012; UAM:Ento:507963. • 1 ♀; Same collection data as previous; 29 July 2012; UAM:Ento:507959. • 1 ♀; Same collection data as previous; 64.82578, -166.00455; 27 July 2012; UAM:Ento:507960. • 1 ♂; Same collection data as previous; 64.82407, -166.00308; 18 July 2012; UAM:Ento:507961. • 1 ♂; Same collection data as previous; 64.82347, -166.00791; 7–13 June 2013; UAM:Ento:250553. • 1 ♂; Same collection data as previous; 64.82705, -166.05324; 14–19 June 2013; Kelly Overduijn, Terri Wild, Skyler Jordan, Mitch Parsons leg.; UAM:Ento:433067. • 1 ♂; Same collection data as previous; 64.8318, -166.03925; 20–25 July 2013; UAM:Ento:433217. • 1 ♀; Same collection data as previous; 64.82349, -166.00785; UAM:Ento:433069. • 1 ♂; Seward Peninsula, Blume Creek; 64.84007, -166.06606; 20–25 June 2013; UAM:Ento:433068. • 1 ♂; Same collection data as previous; 64.82337, -166.00773; UAM:Ento:508000.

##### Diagnosis.

*Boreus timaryi* can be differentiated from all other *Boreus* species by the presence of one short bristle on each side of the posterior edge of the pronotum, adjacent to the forewings and the lack of bristles on the anterior edge of the pronotum, and the presence of tergal apophyses on the 3^rd^ and 4^th^ abdominal segments in males.

##### Description.

***Head***. Head dark, metallic blue-bronze from top of head to just below eyes, fading to pale reddish-brown at tip of rostrum. Head length measured from top of head to distal tip of labrum 1.37–1.82 mm (female mean = 1.82, male mean = 1.56). Eyes reddish to purplish-gray. Diameter of eye measured parallel to direction of head 0.44–0.62 mm (female mean = 0.56, male mean = 0.51). Lateral ocelli nearly touching compound eyes. Median ocellus present between base of antennae. Antennae with 19–21 antennomeres (female mode = 19, male mode = 21). Antennae brown, paler basally, darker distally. Fine white pubescence covering entire head, sparse and short except on frontal region and along median edge of rostrum where hairs are noticeably denser and longer. Maxillolabial complex to rostrum length ratio 0.80–0.93 (female mean = 0.85, male mean = 0.86).

***Thorax***. Pronotum metallic brown to blue-bronze, with two indistinct transverse furrows. Anterior edge of pronotum without bristles. Posterior edge of pronotum usually with one lateral bristle on each side, adjacent to attachment point of median margin of forewings. Meso- and metanotum reddish-brown, less metallic than pronotum, without bristles.

***Legs***. Leg segments orangish-brown, darkened adjacent to joints. Coxae covered in long, white pilosity. All other leg segments covered in short, fine, dark hairs. Femur with stout black apical-femoral spine, without additional spines. Tibia with two orange apical spurs. Tibia and tarsi with two rows of dark spines on ventral surface. Tibia with 2–5 short black spines on dorsal surface. Tarsi without spines on dorsal surface.

***Female wings***. Forewings orange, translucent, covered in short, sparse, fine, dark hairs; reduced to small ovals 0.52–0.6 mm (mean = 0.57) in length, covering entire hindwing.

***Male wings***. Forewings orangish-brown, similar in color to legs. Forewing length 1.42–1.84 mm (mean length = 1.68). Outer margin of forewing narrows noticeably near mid length when viewed dorsally. Margins of forewings bear 29–42 (mode = 33) inner spines and 5–16 (mode = 9) outer spines. Terminal spine of forewings large, brown to black. Hindwings similar in length to forewings, ending in flattened, curled tips. Midlength of hindwings with 6–16 (mode = 13) spines.

***Female abdomen***. Terga metallic blue-green-bronze. Sterna similar in color to terga, but less metallic. Terga and sterna covered in short, fine, sparse, near-translucent hairs. Intersegmental membrane varies in color from yellow to pink. Ovipositor (segments 9–11) orange, darker distally, 1.70–1.90 mm (mean = 1.80) in length. Ovipositor to rostrum length ratio 1.40–1.65 (mean = 1.53). Tergum 8 with fine wrinkles and short, fine, sparse, near-translucent hairs. Tergum 9 0.30–0.37 mm (mean = 0.343) in length, with short, fine, sparse, and near-translucent hairs. Tergum 10 1.12–1.20 mm (mean = 1.16) in length, without hairs. Segment 11 and cerci fused, 0.33–0.45 mm (mean = 0.41) in length, with sparse, short translucent hairs. Ventral ovipositor valves (valvulae) 1.40–1.70 mm (mean = 1.50) in length, usually noticeably shorter than ovipositor. Valvulae with fine, translucent hairs on the basal half of the ventral surface and short, peg-like spines on the distal third.

***Male abdomen***. Terga and sterna 1–8 colored as in female. Terga 3 and 4 with raised tergal apophyses along dorso-posterior margin, most noticeable on tergum 3. Tergum 8 and sternum 8 almost entirely fused. Tergum 9 and sternum 9 not fused. Tergum 9 with low hood, clefted by median septum. Tergal pockets covered in dense, long white hairs, with short spines on lateral edges. Sternum 9 (hypandrium) entire, without cleft, reaches base of dististyles. Dististyles brown, darkening to black distally. Basal lobe of dististyle square-tipped, separated from base of dististyle by slim cleft. Dististylar claw long and pointed. Dististyle with few, elongated, thin denticles along outer margin between claw and basal lobe. Tergum 10 and sternum 10 reduced, small oval sclerites, covered in short, fine hairs, rarely visible.

***Body length***. Females: preserved in ethanol 5.40–7.49 mm (mean = 6.51). Males: preserved in ethanol 2.84–5.33 mm (mean = 4.02).

***Variation***. As in *B. westwoodii* and *B. hyemalis*, the tergal apophyses are highly variable in *B. timaryi*. The apophysis on tergum 3 is present in all individuals, but varies in shape from anvil-like to square. The apophysis on tergum 4 is reduced and nearly absent in some individuals. No consistent differences in morphology were noted between specimens collected in June versus those of July.

***DNA***. We obtained six COI sequences for *B. timaryi* from three localities. There were six variable sites within the sequences. BIN: BOLD:ACW5335. Holotype sequence: UAMIC4039-21.

##### Etymology.

The specific epithet is named for Timary Stenek of Shishmaref, who, at age 13, collected a specimen of this species when participating in a Bering Land Bridge National Preserve Bioblitz event in 2016. This species is named for Timary’s contribution to science, in celebration of the role of citizen science in the documentation of biodiversity, and the past and continuing role of Alaska Native communities in stewarding wildlife and biodiversity.

##### Distribution.

*Boreus timaryi* has been collected from two areas on the Seward Peninsula in western Alaska (Fig. 10B). Specimens were collected from two sites outside of Nome, Alaska (Blume Creek, Feather River), and from a third site approximately 125 km northeast of Nome in the Bering Land Bridge National Preserve.

##### Ecology.

*Boreus timaryi* has been collected in subarctic tundra regions. The two *B. timaryi* specimens that were hand collected in the Bering Land Bridge National Preserve were found under rocks in a mossy area bordering a small snowfield (Fig. 10C). The remaining specimens were collected in pitfall traps placed near plover nests in an area dominated by willow, alder, and dwarf birch with groundcover consisting of mosses, grasses, and lichens. *Boreus timaryi* has only been collected in June and July and is the second Nearctic *Boreus* species to be documented as summer active, the first being *B. borealis* (Banks 1923).

The sex ratios of *B. timaryi* are approximately equal, with a total of seven ♂♂ and six ♀♀ collected. All female specimens were collected in July, while four male specimens were collected in June and three were collected in July. However, this pattern may be an artifact of the small sample size and further sampling efforts are needed to understand the phenology of this species.

##### Remarks.

This is the first documented Nearctic species that has tergal apophyses, which had previously been assumed to be present only in European species, including notably *B. hyemalis* and *B. westwoodii*. Unlike *B. hyemalis* and *B. westwoodii*, *B. timaryi* has tergal apophyses on the third and fourth abdominal segments, as opposed to the second and third abdominal segments. The presence of this character differentiates *B. timaryi* from all other *Boreus* species.

#### Boreus gracilis

Taxon classificationAnimaliaMecopteraBoreidae

Carpenter, 1935, stat. res.

6BA4DE3C-FB6B-5093-9505-E6030441AD84

Figs 1F, 2G, 2H, 11, 21A, 22A, 26A

##### Material examined.

Redescription based on 1 ♂ in ethanol, 3 ♂♂ pinned, 6 ♀♀ in ethanol, and 8 ♀♀ pinned: ***Paratype*** United States – **Alaska** • ♀; Between Kennicott and McCarthy; 61.4, -142.9; 15 Apr. 1934; Wilbur Lloyd leg.; MCZ:Ent:22361.

##### Other material.

(4 ♂♂, 13 ♀♀) United States – **Alaska** • 3 ♂♂, 7 ♀♀; Same collection data as paratype; 15–29 Apr. 1935; J. E. Lloyd leg.; MCZ:Ent:732346, MCZ:Ent:732347, MCZ:Ent:732348, MCZ:Ent:732349, MCZ:Ent:732350, MCZ:Ent:732351, MCZ:Ent:732352, MCZ:Ent:732353, MCZ:Ent:732354, MCZ:Ent:732355. • 1 ♀; Wrangell-St. Elias National Park, along Wagon Road between McCarthy and Kennicott; 61.47138889, -142.87944444; 16 Sept. 2021; Thalles Pereira leg.; UAM:Ento:451096. • 4 ♀♀; Same collection data as previous; 61.47305556, -142.88027778; TLK leg.; UAM:Ento:451105, UAM:Ento:457835, UAM:Ento:457836, UAM:Ento:457837. • 1 ♂, 1 ♀; Same collection data as previous; 61.4690, -142.8776; 23 Sept. 2022; UAM:Ento:477811, UAM:Ento:477815.

##### Diagnosis.

*Boreus gracilis* can be differentiated from non-Alaskan members of the *brumalis* subgroup of the *nivoriundus* species group sensu Penny 1977 (*B. brumalis*, *B. bomari*, *B. insulanus*, *B. nix*, and *B. pilosus*) by the greatly reduced or absent median ocellus, and from most members of the species group by the presence and relative length of cruciate bristles on the female mesonotum, which are similar in length to pronotal bristles and distinctly longer than the surrounding hairs. *Boreus gracilis* differs from its apparent sister species (see below) *B. tananaensis* by the length of the thoracic and abdominal hairs, which reach up to half the length of the thoracic bristles in *B. gracilis* and are shorter than half the length of the thoracic bristles in *B. tananaensis*. *Boreus gracilis* can also be differentiated from *B. tananaensis* by coloration; *B. gracilis* is brown with a gold iridescence while *B. tananaensis* is brown with a green-blue iridescence. This difference in coloration is less obvious in pinned specimens. Male *B. gracilis* also differ from *B. tananaensis* in the shape of the cleft of the distal hood, which is long and narrow as opposed to short and triangular in *B. tananaensis*, and the posterior margin of the denticular area, which is straight in *B. gracilis* and curves in towards the cleft in *B. tananaensis*.

##### Description.

***Head***. Head dark with golden iridescent sheen from top of head to below eyes. Maxillolabial complex non-iridescent, similar in color to rest of head except on the lateral sides of the clypeus, which are paler. Head length measured from top of head to distal tip of labrum 1.14–1.57 mm (female mean = 1.42, male mean = 1.22). Eyes reddish-purple. Length of eye 0.42–0.54 mm (female mean = 0.47, male mean = 0.43). Lateral ocelli more than diameter of ocellus away from compound eye margin. Median ocellus reduced, significantly smaller than lateral ocelli, or absent. Epistomal suture extending laterally to intersect the margin of the maxillolabial complex, creating a triangular shape. Antennae with 20–22 antennomeres (mode for both sexes = 21). Antennae dark brown. Fine white hairs covering entire head, most dense and long on frontal region. Maxillolabial complex to rostrum length ratio 0.59–0.93 (female mean = 0.85, male mean = 0.76).

***Thorax***. Pronotum grayish-brown, paler than head and abdominal terga, with two transverse furrows. Anterior edge of pronotum with 2–4 (mode = 3) bristles on each side. Posterior edges of pronotum with 2–3 (mode = 2) bristles on each side. Mesonotum similar in color to pronotum, episternum and epimeron paler than dorsal face. Mesoscutellum with two bristles curving inward in females, males without bristles. Metanotum similar in color to mesonotum, without bristles.

***Legs***. Leg segments grayish-brown, darker adjacent to joints, giving a striped appearance. Coxae covered in long, fine, sparse, white hairs, denser on anterior face. All other leg segments covered in short, fine, dark hairs. Femur with apical-femoral spine present, long, pointed, and black, without additional spines. Tibia with two long, translucent-brown apical spurs. Tibia and tarsi with two rows of yellow spines on ventral surface, no spines on dorsal surface.

***Female wings***. Forewings grayish-brown, translucent, covered in short, dense, fine, dark hairs, reduced to small ovals 0.30–0.41 mm (mean = 0.37) in length, covering entire hindwing.

***Male wings***. Forewings grayish-brown, similar in color to legs, covered in short, dense, fine, dark hairs. Forewing length 1.38–1.48 mm (mean = 1.43). Outer margin of forewing tapers evenly when viewed dorsally. Margins of forewings bear 15–19 (no mode) inner spines and 13–16 (mode = 13) outer spines. Terminal spine of forewing significantly longer than penultimate inner spine. Hindwings longer than forewings, ending in flattened, curled tips. Hindwings with seven or eight (mode = 7) peg-like spines which decrease in size distally. Distal half of hindwings with dense, short, white hairs, ending before curved tip.

***Female abdomen***. Terga and sterna dark grayish-brown with gold iridescent sheen, covered in long, fine, dense, near-translucent hairs. Intersegmental membrane varies in color from red to pale yellow. Tergum 8 with fine wrinkles and short, fine, sparse, near-translucent hairs. Female terminalia (segments 9–11) greyish brown, similar in color to legs. Ovipositor (segments 9–11) 1.03–1.31 mm (mean = 1.20) in length. Ovipositor to rostrum length ratio 1.19–1.50 (mean = 1.37). Tergum 9 0.08–0.18 mm (mean = 0.13) in length, with long, fine, sparse, near-translucent hairs. Tergum 10 0.69–0.85 mm (mean = 0.80) in length, with sparse, long hairs present. Segment 11 and cerci fused, 0.30–0.39 mm (mean = 0.35) in length, with short, fine, sparse translucent hairs on dorsal surface and longer, thicker, denser, translucent-white hairs on ventral surface. Ventral ovipositor valves (valvulae) 0.89–1.20 mm (mean = 1.07) in length, with medium-length, fine, sparse, translucent hairs on the ventral surface and short, peg-like spines with hairs emerging from them on the distal half to third.

***Male abdomen***. Terga and sterna 1–8 colored as in female. Tergum 8 and sternum 8 not fused. Tergum 9 and sternum 9 not fused. Tergum 9 with a small hood extending laterally to median of the denticular areas, with short median septum and deep cleft. Cleft margins parallel. Tergal pockets not fully separated, covered in fine, short white hairs. Denticular area does not extend into tergal pockets. Sternum 9 (hypandrium) is broadly triangular, with distal edge deeply notched, reaching to distal edge of basistyles, covered in long, fine, dense, white hairs. Dististyles dark brown, covered in long, fine, dense, white hairs. Basal lobe of dististyle square, separated from base of dististyle by triangular cleft. Outside of dististyle with denticles extending from base of dististylar claw to basal lobe. Tergum 10 and sternum 10 reduced, small oval sclerites, covered in short, fine hairs, rarely visible.

***Body length***. Females: preserved in ethanol 3.77–4.54 mm (mean = 4.21), pinned 3.81–4.81 mm (mean = 4.355). Males: preserved in ethanol 3.09 mm, pinned 2.38–3.27 mm (mean = 2.82).

***DNA***. We obtained three COI sequences for *B. gracilis* from one locality. There were no variable sites within the sequences. BOLD BIN: BOLD:AES2569.

##### Distribution.

*Boreus gracilis* has been collected between McCarthy and Kennicott Alaska (Fig. 11B). In 1934, *B. gracilis* was collected between McCarthy and Kennicott (Carpenter 1935), and in 1935 additional specimens were collected in Kennicott and in McCarthy (Carpenter 1936). In 2021 and 2022, specimens were collected between McCarthy and Kennicott along the Wagon Road trail.

##### Ecology.

*Boreus gracilis* has only been collected in sympatry with *B. intermedius*. Specimens were collected from boreal forest in a mountainous area bordering Kennicott glacier, consisting of predominantly spruce, alder, and willow, and groundcover consisting of moss and short shrubs (Fig. 11C). Within this habitat, *B. gracilis* specimens were collected from moss on steep, near vertical rock surfaces and glacial deposits. In 1934 and 1935, this species was collected in April. Despite efforts to collect in the spring (May), *B. gracilis* has since only been collected in September (2021, 2022). Specimens were collected on overcast and rainy days when the moss cover was very wet. In 1934, *B. gracilis* specimens were collected on top of snow.

Both historic (1934: 2 ♀♀, 1935: 3 ♂♂, 7 ♀♀) and recent (2021: 5 ♀♀, 2022: 1 ♂, 1 ♀) collection events indicate that female *B. gracilis* are more numerous than males (total: 4 ♂♂, 15 ♀♀). This is the only Alaskan species in which such an uneven sex ratio has been observed, but similar ratios have been observed in other species (Lestage 1941; Cooper 1974) and larger sample sizes are needed to be confident of this result.

##### Remarks.

As the holotype of *B. gracilis* has been lost, we were only able to examine a paratype. The *B. gracilis* specimens collected in 1934 were initially identified as *B. unicolor* (Lloyd 1934), a species that has since been synonymized under *B. californicus* Packard, 1870 (Penny 1977). Contrary to what Lloyd reported (1934), the morphology of the *B. gracilis* specimens is inconsistent with the description of *B. unicolor*, both in terms of overall size, ovipositor length, and pilosity.

The original description for *B. gracilis* was based on female specimens only. Carpenter (1935) identified the female forewings not entirely covering the hindwing scar as a diagnostic character; however, this appears to be an artifact of preservation – this trait is not found in any *B. gracilis* specimen stored in alcohol or freshly collected. In his description of male *B. gracilis*, Carpenter (1936) noted that males had “nearly straight outer margins of the wings.” Penny (1977) used this trait as the basis for his synonymization of *B. gracilis* under *B. nix*: “[Carpenter] mentioned that this species differed from *B. nix* only in having male wings which were not abruptly narrowed at mid-length. However, in observing the series he used in describing the male wings, I noted that these specimens do have such abruptly narrowed wings. As there are no other differences, I consider *gracilis* as a junior synonym of *nix*.” In reviewing the three male specimens remaining from Carpenter’s original series of male *B. gracilis* as well as one freshly collected male specimen, the wings appear to taper evenly, as was described by Carpenter. This, paired with the presence of a median septum in the tergal hood of males, the consistent presence of mesothoracic bristles on females, and the absence or near-absence of a median ocellus supports the validity of the specific status of *B. gracilis*, as do COI data (see molecular results section below). We therefore resurrect *Boreus gracilis* in this work. The collection data on the label of all specimens collected in 1935 reads “Kennecott, Alaska April 15, ‘35.” However, Carpenter (1936) stated of these specimens “1 ♀ was collected at Kennecott, Alaska, April 15, 1935; and the rest at McCarthy, Alaska, April 29, 1935.” It is unclear which specimens were collected at which locality and on which date.

#### Boreus borealis

Taxon classificationAnimaliaMecopteraBoreidae

Banks, 1923

CED8F158-E1C6-517B-A98B-0585A7CF35F6

Figs 1A, 1B, 2A, 2B, 12, 19A, 23B, 24C, 24D, 25B, 27B, 28B

##### Material examined.

Redescription based on 6 ♂♂ in ethanol, 2 ♂♂ pinned (including the lectotype), 7 ♀♀ in ethanol, and 1 ♀ pinned: ***Lectotype***. United States – **Alaska** • ♂; St. Paul Island, grassy bank, beyond village wells; 57.128, -170.274; 23 May 1914; Alvin G. Whitney and Elsie G. Whitney leg.; MCZ:Ent:11277.

##### Other material.

(7 ♂♂, 8 ♀♀) United States – **Alaska** • 1 ♂, 1 ♀; St. Paul Island; 57, -170; July-Aug. 1925; A. Christofferson leg.; CASENT8076679. • 1 ♂, 1 ♀; St. George Island, harbor; 56.56951, -169.6664; 8 Aug. 2012; DSS and Casey Engstrom leg.; UAM:Ento:235219, UAM:Ento:235218. • 2 ♂♂; St. George Island; 56.594966, -169.55435; 8 Aug. 2012; DSS and Matt Klosterman leg.; UAM:Ento:504854, UAM:Ento:261148. • 2 ♂♂, 5 ♀♀; St. Paul Island, Kamanista Quarry; 57.16001, -170.26517; 30 July 2022; DSS and TLK leg.; UAM:Ento:476192, UAM:Ento:476193, UAM:Ento:476194, UAM:Ento:476195, UAM:Ento:476196, UAM:Ento:476197, UAM:Ento:476257. • 1 ♂, 1 ♀; same collection data as previous; 31 July 2022; UAM:Ento:476191, UAM:Ento:476259.

##### Diagnosis.

Both sexes of *B. borealis* can be differentiated from non-Alaskan members of the *californicus* species group sensu Penny 1977 (*B. californicus*, *Boreus coloradensis* Byers, 1955) by the color of the thoracic segments and legs, which are orange and significantly paler than the abdominal segments in both pinned and alcohol preserved specimens. Female *B. borealis* can be differentiated from its apparent sister species (see below) *B. intermedius* by the lack of hairs emerging from the peg-like spines at the distal end of the ovipositor. Male *B. borealis* can be differentiated from other members of the *californicus* species group, including *B. intermedius*, by the lack of spines on the ventral surface of the hind wings.

##### Description.

***Head***. Top of head dark with yellow-green-blue iridescent sheen from top of head to below eyes, fading to orange on the maxillolabial complex. Head length measured from top of head to distal tip of labrum 1.47–1.98 mm (female mean = 1.82, male mean = 1.59). Eyes red to reddish-purple. Diameter of eye measured parallel to direction of head 0.53–0.63 mm (female mean = 0.59, male mean = 0.55). Lateral ocelli about diameter of ocellus away from compound eye margin. Median ocellus present between base of antennae, noticeably smaller than lateral ocelli. Antennae with 18–20 antennomeres (mode for both sexes = 20). Antennae brown, fading to dark brown or black distally. Fine white pubescence covering entire head, sparse except on frontal region, where hairs are noticeably denser and longer. Maxillolabial complex to rostrum length ratio 0.81–0.95 (female mean = 0.85, male mean = 0.88).

***Thorax***. Pronotum brown, less iridescent than head and abdominal terga, with one transverse furrow. Anterior and posterior edges of pronotum without bristles. Meso- and metanotum brown, paler than pronotum, without bristles.

***Legs*** orange, darkened adjacent to joints. Coxae covered in short, fine, sparse, white hairs. All other leg segments covered in short, fine, dark hairs. Femur with apical-femoral spine present, short, pointed, and black, without additional spines. Tibia with two long, translucent-brown apical spurs. Tibia and tarsi with two rows of dark spines on ventral surface, no spines on dorsal surface.

***Female wings***. Forewings yellowish-orange, translucent, covered in fine dark hairs, reduced to small ovals 0.50–0.66 mm (mean length = 0.55 mm) in length, covering entire hindwing.

***Male wings***. Forewings yellowish orange, similar in color to legs, covered in short, dark hairs. Forewing length 1.64–1.96 mm (mean length = 1.80). Outer margin of forewing narrows noticeably near mid length when viewed dorsally. Margins of forewings bear 31–48 (mode = 39) inner bristles and 8–14 (mode = 12) outer spines. Terminal spine of forewings significantly longer than penultimate inner forewing spine, brown. Hindwings similar in length to forewings, ending in flattened, curled tips. Hindwings without spines. Distal half of hindwings with dense, short white hairs, ending before curved tip.

***Female abdomen***. Terga and sterna dark with blue-green-yellow iridescence, covered in short, fine, sparse, near-translucent hairs, denser on sterna than on terga. Intersegmental membrane varies in color from red to pale yellow. Tergum 8 with fine wrinkles and short, fine, sparse, near-translucent hairs. Female terminalia (segments 9–11) orange, darker than legs. Ovipositor (segments 9–11) 1.40–1.81 mm (mean = 1.59) in length. Ovipositor to rostrum length ratio 1.32–1.67 (mean = 1.41). Tergum 9 0.15–0.35 mm (mean = 0.25) in length, with short, fine, sparse, near-translucent hairs. Tergum 10 0.83–1.05 mm (mean = 0.95) in length, without hairs. Segment 11 and cerci fused, 0.45–0.55 mm (mean = 0.50) in length, with short, fine, sparse translucent hairs on dorsal surface and longer, thicker, denser, translucent-white hairs on ventral surface. Ventral ovipositor valves (valvulae) 1.10–1.41 mm (mean = 1.22) in length, with medium-length, fine, sparse, translucent hairs on the ventral surface and short, peg-like spines on the distal third of the ventral surface.

***Male abdomen***. Terga and sterna 1–8 colored as in female. Tergum 8 and sternum 8 fused.

Tergum 9 and sternum 9 not fused, more brown than previous segments. Tergum 9 with hood extending laterally to edge of denticular area, clefted by median septum which extends slightly beyond hood. Tergal pockets covered in dense, fine, long white hairs. Denticular area does not extend into tergal pockets. Sternum 9 (hypandrium) is broadly triangular, with distal edge entire and square, reaching to distal edge of basistyles, covered in long, fine, dense, white hairs. Dististyles orange except on basal lobe and claw, which are black and shiny, covered in long, fine, dense, white hairs, except on basal lobe and claw. Basal lobe of dististyle pointed, separated from base of dististyle by triangular cleft. Inside of dististyle with small, black denticles extending between dististylar claw and basal lobe. Outside of dististyle with denticles extending from base of dististylar claw to below basal lobe. Tergum 10 and sternum 10 reduced, small oval sclerites, covered in short, fine hairs, rarely visible.

***Body length***. Females: preserved in ethanol 5.54–6.68 mm (mean = 6.01), pinned 7.94 mm. Males: preserved in ethanol 3.48–5.16 mm (mean = 4.01), pinned 3.64–4.88 mm (mean = 4.259).

***DNA***. We obtained five COI sequences for *B. borealis* from two localities. There was one variable site within the sequences. BIN: BOLD:ACG1116.

##### Distribution.

*Boreus borealis* has been collected in the Pribilof Islands, Alaska on St. Paul Island and St. George Island (Fig. 12B). In 1914, this species was collected from two localities on St. Paul Island, both adjacent to the town of Saint Paul. In 2022, specimens were collected from Kamanista Quarry, near the middle of St. Paul Island. Specimens collected on St. Paul Island in 1925 and 1975 did not have specific localities recorded. In 2012, *B. borealis* was collected from two localities on St. George Island, one near the docks and one inland.

##### Ecology.

The Pribilof Islands are made up of subarctic mesic maritime tundra. The area is treeless, and consists of a mixture of coastal sand dunes, cliffs, extinct cinder cones and lava flows, bogs, marshes, and grass meadows. In 1914, two specimens were collected on St. Paul Island from mosses in a grassy bank outside the town of Saint Paul and two additional specimens were found on sand dunes in a nearby area. In 2012, *B. borealis* was collected on St. George Island under a rock in an area with *Lupinus*, *Angelica*, and mosses. Two additional specimens were collected dead in a shallow puddle under debris. In 2022, nine *B. borealis* specimens were collected from St. Paul Island along north-west facing mossy cliffs in Kamanista Quarry (Fig. 12C) in a mixture of star mosses and feather mosses. Collecting efforts in other areas of the quarry and island were unsuccessful. *Boreus borealis* have only been collected in May, July, and August, however, there has been limited if any sampling done on the Pribilof Islands outside of the summer as the island is difficult to reach and often has inclement winter weather. *Boreus borealis* apparently has an approximately equal sex ratio. In total, 9 ♂♂ and 10 ♀♀ have been collected from this species.

##### Remarks.

The original description of *B. borealis* was based on four specimens, two males and two females. The species was diagnosed based on the color of the coxae and pleura, the length of the wings, and the overall size. The description of the color of the coxae and pleura is consistent with *B. intermedius*, and as noted by Carpenter (1931) and Penny (1977), the size of the wings is similar to some other *Boreus* species. While *B. borealis* are on average larger than *B. californicus*, *B. coloradensis*, and *B. intermedius*, the ranges in body size overlap between species too much for this to be a useful diagnostic character. Penny’s morphological description of *B. borealis* was based on one of the syntypes (which he assigned as the lectotype), one additional male and one additional female. Penny stated that “...males can be identified by the large number of setae lining the tergal hood. Females cannot be positively identified, except by locality. Banks (1923) mentioned that this was the only species with pale coxae and pleura, but some individuals of *B. californicus* and *pilosus* also have pale coxae and pleura.” However, the color difference between the coxae and pleura and the abdominal segments is far greater and more obvious in *B. borealis* and *B. intermedius* than in any other species we have seen, and as noted above female specimens can be identified to species by the combination of the color of the thorax and legs, and the absence of hairs emerging from the spines at the distal end of the ovipositor. Otherwise, Penny’s (1977) description of *B. borealis* is generally accurate, but lacks information on variation due to the limited number of specimens studied.

#### Boreus intermedius

Taxon classificationAnimaliaMecopteraBoreidae

Lloyd, 1934

F65AFAED-5989-5AE2-97FD-EA53ADDA1CA1

Figs 1E, 2C, 2D, 13, 14, 24A, 24B, 25A, 27A, 28A

##### Material examined.

Redescription based on 6 ♂♂ in ethanol, 1 ♂ pinned, 9 ♀♀ in ethanol, 3 ♀♀ pinned: United States – **Alaska** •1 ♂, 3 ♀♀; Between McCarthy and Kennicott; 61.4, -142.9; 15 Apr. 1935; J. E. Lloyd leg.; MCZ:Ent:732356, MCZ:Ent:732357, MCZ:Ent:732358, MCZ:Ent:732359. • 2 ♂♂, 2 ♀♀; Wrangell-St. Elias National Park, along Wagon Road between McCarthy and Kennicott; 61.45381, -142.88228; 13 May 2021; DSS, TLK and Thalles Pereira leg.; UAM:Ento:447163, UAM:Ento:447165, UAM:Ento:447167, UAM:Ento:447168. • 1 ♀; Same collection data as previous; 61.45427, -142.88199; UAM:Ento:447164. • 1 ♀; Same collection data as previous; 61.45426, -142.88248; UAM:Ento:447166. • 1 ♂, 1 ♀; Same collection data as previous; 61.45935, -142.88072, 15 Sept. 2021; TLK, Thalles Pereira, and Hannah Doerrier leg.; UAM:Ento:451000, UAM:Ento:451067. • 1 ♀; Same collection data as previous; 61.45417, -142.88203; UAM:Ento:451001. • 2 ♀♀; Same collection data as previous; 61.475, -142.8811; 16 Sept. 2021; UAM:Ento:451065, UAM:Ento:451066. • 1 ♂; Same collection data as previous; 61.47138889, -142.87944444; UAM:Ento:451103. • 1 ♂; Same collection data as previous; 61.47416667, -142.88055556; UAM:Ento:451104. • 1 ♀; Same collection data as previous; 61.47306, -142.8803; 23 Sept. 2022; TLK leg.; UAM:Ento:477814. • 1 ♂; Same collection data as previous; 61.4690, -142.8776; 24 Sept. 2022; UAM:Ento:477812.

**Figure 13.**
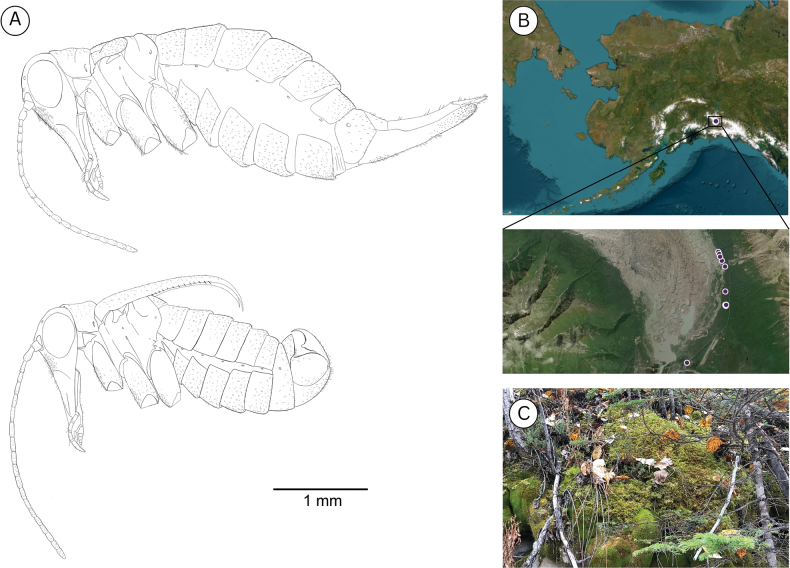
**A**. *Boreus intermedius* female (top) and male (bottom) viewed laterally. **B**. Collection localities for *B. intermedius*. **C**. Habitat of *B. intermedius* (photo credit: TLK).

**Figure 14.**
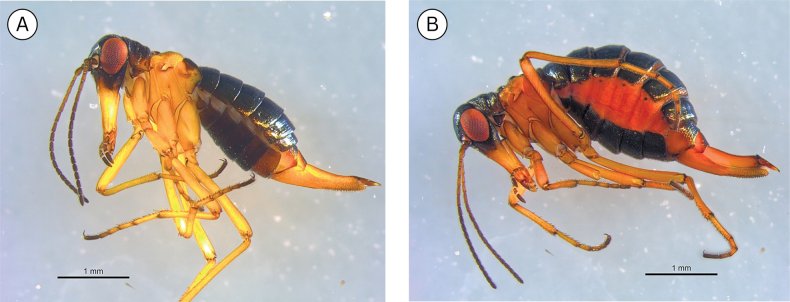
Size and color difference in *B. intermedius* specimens collected in the: **A**. Fall (15 September 2021, UAM:Ento:451067) and **B**. Spring (13 May 2021, UAM:Ento:447163). Photo credits: TLK.

##### Diagnosis.

Both sexes of *B. intermedius* can be differentiated from non-Alaskan members of the *californicus* species group sensu Penny 1977 (*B. californicus*, *B. coloradensis*) by the color of the thoracic segments and legs, which are significantly paler than the abdominal segments in both pinned and alcohol preserved specimens. Female *B. intermedius* can be differentiated from its apparent sister species (see below) *B. borealis* and other members of the *californicus* species group by the presence of hairs emerging from the peg-like spines at the distal end of the ovipositor, which are absent in these other species. Male *B. intermedius* can be differentiated from *B. borealis* by its short tergal hood septum, which does not reach the base of the denticular area.

##### Description.

***Head***. Top of head dark, iridescent yellow-green from top of head to below eyes, fading to yellow or orange on the maxillolabial complex. Head length measured from top of head to distal tip of labrum 1.28–1.57 mm (female mean = 1.51, male mean = 1.39). Eyes reddish-orange to reddish-purple. Diameter of eye measured parallel to direction of head 0.44–0.55 mm (female mean = 0.52, male mean = 0.49). Lateral ocelli approximately one ocellus away from compound eye margin. Median ocellus present between base of antennae, noticeably smaller than lateral ocelli. Antennae with 19–22 antennomeres (mode for both sexes = 20). Antennae brown, fading to dark brown distally. Fine white hairs covering entire head, sparse and short except on frontal region, where hairs are noticeably denser and longer. Maxillolabial complex to rostrum length ratio 0.74–0.89 (female mean = 0.83, male mean = 0.84).

***Thorax***. Pronotum dark and iridescent yellow-green, similar in coloration to head and abdominal terga, with one transverse furrow. Anterior and posterior edges of pronotum without bristles. Meso- and metanotum similar in color to pronotum, without bristles.

***Legs***. Leg segments yellow-orange, darkened adjacent to joints. Coxae covered in short, fine, sparse, white hairs. All other leg segments covered in short, fine, sparse, dark hairs. Femur with apical-femoral spine present, short, pointed, and black, without additional spines. Tibia with two long, translucent-brown apical spurs. Tibia and tarsi with two rows of dark spines on ventral surface, no spines on dorsal surface.

***Female wings***. Forewings yellowish-orange, paler than legs, translucent, covered in fine dark hairs, reduced to small ovals 0.41–0.50 mm (mean = 0.45) in length, covering entire hindwing.

***Male wings***. Forewings yellowish-orange, paler than legs, translucent, covered in fine dark hairs. Outer margin of forewings noticeably narrowed near mid length when viewed dorsally. Forewing length 1.59–1.91 mm (mean = 1.74). Margins of forewings bear 25–32 (no mode; each observed value occurred only once) inner spines and 6–14 (mode = 11) outer spines. Forewing spines along both margins curl in towards margin and increase in length distally. Terminal spine of forewing less than twice as long as penultimate inner forewing spine, brown. Hindwings similar in length to forewings, ending in flattened, curled tips. Hindwing spines number 2–8 (mode = 3) and are significantly smaller than forewing spines. Distal half of hindwings with dense, short white hairs, ending before curved tip.

***Female abdomen***. Terga and sterna 1–7 dark brown and iridescent, covered in short, fine, sparse, near-translucent hairs, denser on sterna than on terga. Intersegmental membrane varies in color from red to pale yellow. Female terminalia (segments 8–11) orange, darker than legs. Tergum 8 with fine wrinkles and short, fine, sparse, near-translucent hairs. Ovipositor (segments 9–11) 1.13–1.33 mm (mean = 1.24) in length. Ovipositor to rostrum length ratio 1.13–1.48 (mean = 1.34). Tergum 9 0.09–0.21 mm (mean = 0.13) in length, with short, fine, sparse, near-translucent hairs. Tergum 10 0.79–0.89 mm (mean = 0.83) in length, with few long, fine sparse, near-translucent hairs. Segment 11 and cerci fused, 0.37–0.41 mm (mean = 0.39) in length, with long, fine, sparse, translucent hairs on dorsal surface and longer, thicker, denser, translucent-white hairs on ventral surface. Ventral ovipositor valves (valvulae) 0.97–1.13 mm (mean = 1.07) in length, with medium-length, fine, sparse, translucent hairs on the ventral surface and short, peg-like spines with taper into fine, hairlike processes pointing posteriorly on the distal third of the ventral surface.

***Male abdomen***. Terga and sterna 1–7 colored as in female. Tergum 8 and sternum 8 fused. Tergum 9 and sternum 9 not fused, more brown than previous segments. Tergum 9 with hood extending laterally to edge of denticular area, clefted by short median septum which extends slightly beyond hood. Tergal pockets covered in dense, fine, long white hairs. Denticular area does not extend into tergal pockets. Sternum 9 (hypandrium) is broadly triangular, with distal edge entire and square, extending beyond the end of basistyles, covered in long, fine, dense, white hairs. Dististyles orange except on basal lobe and claw, which are black and shiny, covered in long, fine, dense, white hairs, except on basal lobe and claw. Basal lobe of dististyle blunt tipped, separated from base of dististyle by triangular cleft. Dististylar claw long and pointed. Outside of dististyle with denticles extending from base of dististylar claw to below basal lobe. Tergum 10 and sternum 10 reduced, small oval sclerites, covered in short, fine hairs, rarely visible.

***Body length***. Females: preserved in ethanol 4.41–5.22 mm (mean = 4.80), pinned 4.26–4.66 mm (mean = 4.50). Males: preserved in ethanol 2.76–3.90 mm (mean = 3.36), pinned 3.89 mm.

***Variation***. *Boreus intermedius* specimens collected in the fall and spring exhibited morphological differences. In specimens collected in the fall (September), the rostrum, legs, wings, meso- and meta-thoracic segments, and terminalia are yellow (Fig. 14A), while in specimens collected in the spring (May), these characters are orange (Fig. 14B). In some fall *B. intermedius* specimens, these characters were also semi-translucent, suggesting that the paler coloration is due to the exoskeleton not being fully developed. The coloration of the terga and sterna is consistent between seasons, and only in one particular teneral specimen were the abdominal segments semi-translucent. The color of the intersegmental membrane and compound eyes also vary seasonally. In spring collected specimens, the intersegmental membrane is pale pink except on the abdomen of females, where it is red, and the eye is reddish-orange. In fall collected specimens, the intersegmental membrane is white to pale yellow, and the eyes are a darker reddish-purple. *Boreus intermedius* also appear to exhibit temporal variation in body size– in both sexes, spring collected specimens were on average 0.5 mm longer than their fall counterparts, though this difference is only statistically significant in females, likely due to the smaller sample size in male specimens (female: fall body length mean = 4.55 mm (*n* = 5), spring body length mean = 5.11 mm (*n* = 4), t-test p-value = 0.0007; males: fall body length mean = 3.21 mm (*n* = 4), spring body length mean = 3.67 mm (*n* = 2) t-test p-value = 0.1819).

***DNA***. We obtained nine COI sequences for *B. intermedius* from four localities. There were four variable sites within the sequences. BIN: BOLD:AEL2631.

##### Distribution.

*Boreus intermedius* has been collected from multiple localities along a 2.4-km section of the Wagon Road Trail between McCarthy and Kennicott, Alaska (Fig. 13B). In 1934, specimens were collected between McCarthy and Kennicott (Lloyd 1934) and in 1935 specimens were collected in McCarthy (Carpenter 1935). In 2021 and 2022 specimens were only found between McCarthy and Kennicott, along the Wagon Road trail. The main road between McCarthy and Kennicott, which was a railroad track bed in the 1930s, was also sampled in spring 2021, but no specimens were found.

##### Ecology.

*Boreus intermedius* has been collected in sympatry with *B. gracilis*, but appears to have a larger range and has also been collected from sites where no *B. gracilis* were found. The specimens were collected from boreal forest in a mountainous area bordering Kennicott glacier, consisting of predominantly spruce, alder, and willow, and groundcover consisting of moss and short shrubs (Fig. 13C). Within this habitat, *B. intermedius* specimens were collected from moss on steep, near vertical rock surfaces and glacial deposits. *Boreus intermedius* has been collected both in the spring (April 1934, April 1935, May 2021) and fall (September 2021, September 2022). Specimens have been collected on sunny, overcast, and rainy days from moss that was very wet and on dry days from moss that was nearly desiccated. In 1934, *B. intermedius* specimens were collected on top of snow.

A total of 10 ♂♂ and 16 ♀♀ *B. intermedius* specimens have been observed. The sex ratio difference could be attributed to the small sample size. Further sampling is needed to test this.

##### Remarks.

The holotype and paratype of this species have been lost. Lloyd (1934) diagnosed *B. intermedius* as “very similar to *B. borealis*. The female agrees perfectly with descriptions of the species (Banks 1923; Carpenter 1931) while the male differs in the shape of the wings which approach the form found in *B. californicus*.” Penny (1977) also noted “males of *B. intermedius* can be separated from other members of the *californicus* group by the septum of the ninth tergal hood, which is short and deeply divided ventrally.” Penny (1977) listed male *B. intermedius* as having no spines on their hind wings, however all specimens we examined had at least two short spines on the ventral surface of each hind wing.

The collection data on the label of all *B. intermedius* specimens collected in 1935 reads “Kennecott, Alaska April 15, ‘35.” Carpenter (1936) stated of these specimens “Three additional specimens (1 ♂, 2 ♂)... have been sent to me by Mr. Lloyd; they were collected at McCarthy, Alaska, April 29, 1935.” Carpenter presumably meant (1 ♂, 2 ♀), but this does not match the number of specimens bearing 1935 collection labels (1♂, 3 ♀♀). It is possible that one of the females with a 1935 label is the missing allotype. If this is true, it is likely specimen MCZ:Ent:732358, which was originally labeled 1934, but the 4 was scratched out and replaced with a 5.

### Phylogenetic results

**Figure 15.**
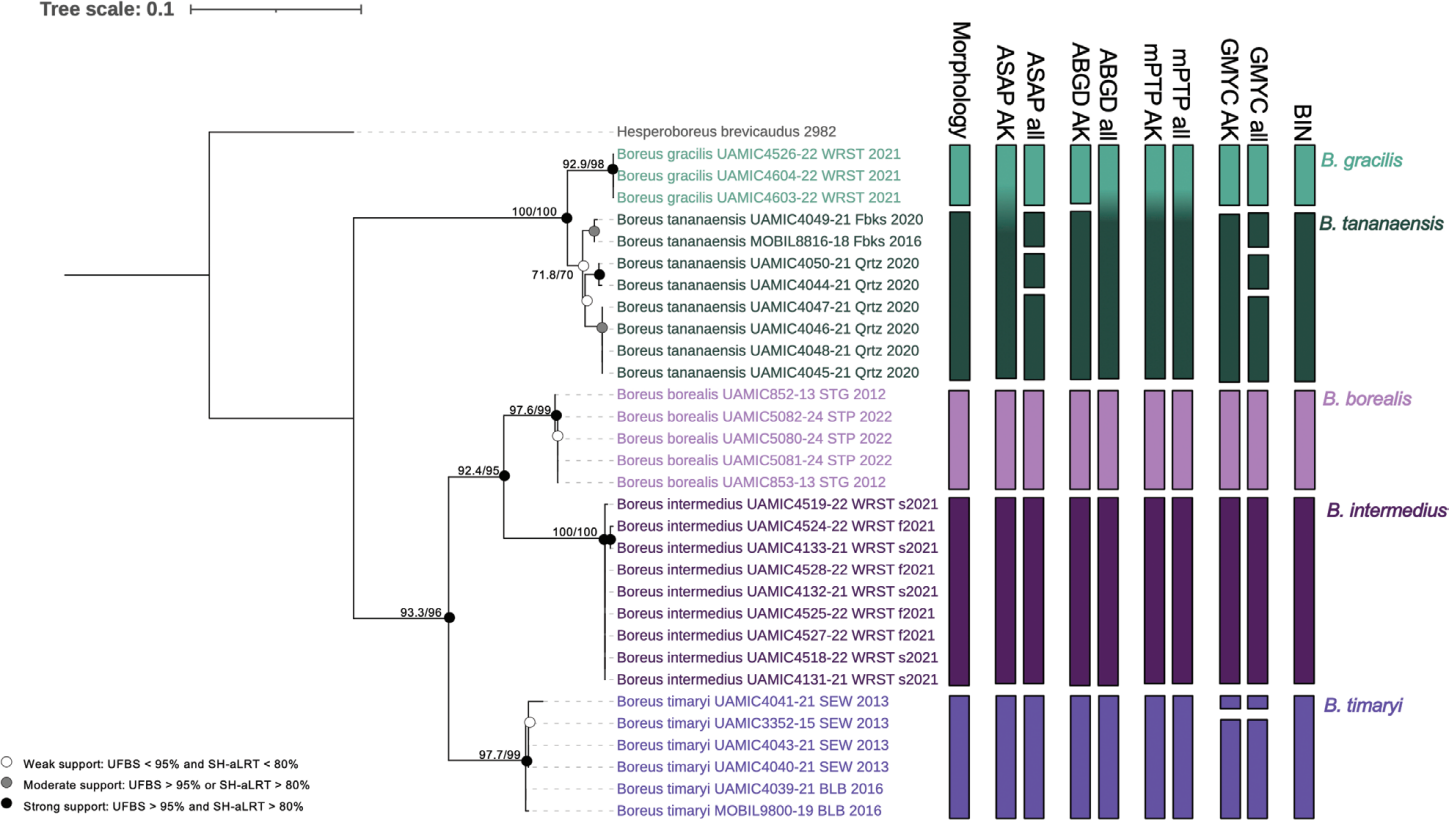
Inferred maximum likelihood phylogeny and species delimitations for Alaskan *Boreus* species. Phylogeny based on the COI barcoding region, estimated in IQ-TREE. We report the results from five species delimitation methods from left to right: morphology (morphological diagnoses), ASAP, ABGD, mPTP, GMYC, and BOLD. For four of the species delimitation methods (ASAP, ABGD, mPTP, and GMYC) two results are reported– the results from the Alaskan *Boreus* dataset (indicated by AK after the species delimitation method name) and also the results from the All *Boreus* dataset pruned to only include the Alaskan *Boreus* species (indicated by all after the species delimitation method name). Branch support is reported as SH-aLRT/UFBS values above the species level. Within species, branch support is indicated by colored circles as either weak (white), moderate (grey), or strong (black). Tip labels include the morphological species identification, followed by the sample code (see Suppl. material 2: table SS1), followed by an abbreviated locality, followed by collection year. For *B. intermedius*, specimens are also marked s (spring) or f (fall). Locality abbreviations: WRST – Between Kennicott and McCarthy in Wrangell St. Elias National Park, Fbks – Fairbanks, Qrtz – Quartz Lake, STP – St. Paul Island, STG – St. George Island, SEW – Seward Peninsula near Nome, BLB – Bering Land Bridge National Preserve.

For the AK *Boreus* dataset, the best fitting model chosen according to BIC was TPM2u+F+G4. In the AK *Boreus* dataset tree (Fig. 15), all Alaskan species were returned as monophyletic, and highly supported (Sh-aLRT ≥ 92.9%, UFBS ≥ 98%), except *B. tananaensis*, which had weak support (SH-aLRT = 71.8%, UFBS = 80%). For the All *Boreus* dataset, the best fitting model chosen according to the BIC was TIM2+F+I+G4. In the All *Boreus* dataset tree all Alaskan species, including *B. tananaensis*, were returned as monophyletic and highly supported (Sh-aLRT ≥ 98%, UFBS ≥ 81%). Outside the Alaskan species, seven species were found to be monophyletic and had strong support (*Boreus elegans* Carpenter, 1935, *Boreus nivoriundus* Fitch, 1847, *Boreus reductus* Carpenter, 1933, *B. bomari*, *B. brumalis*, *B. pilosus*, and *B. semenovi*), one species was found to be monophyletic but had weak support (*B. nix*), and four species were not found to be monophyletic (*B. californicus*, *B. coloradensis*, *B. hyemalis*, *B. westwoodii*).

**Figure 16.**
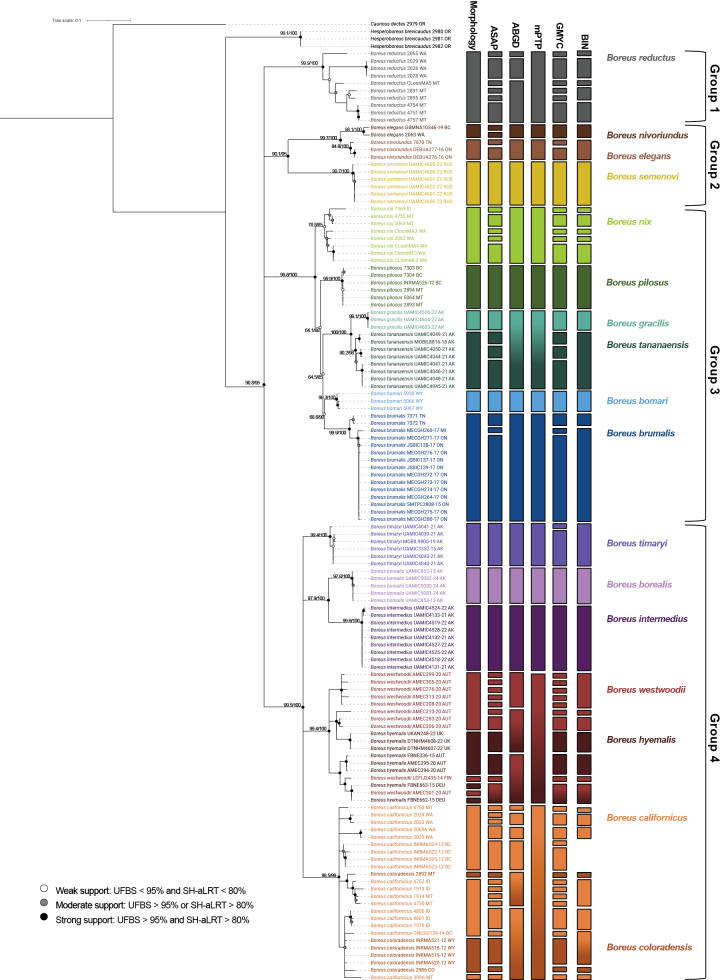
Inferred maximum likelihood phylogeny and species delimitations for All *Boreus* species dataset. Phylogeny based on the COI barcoding region, generated in IQ-TREE. Branch support is reported as SH-aLRT/UFBS values above the species level. Within species, branch support is indicated by colored circles as either weak (white), moderate (grey), or strong (black). Species delimitation methods from left to right: morphology (morphological identification of specimens), ASAP, ABGD, mPTP, GMYC, and BIN. Tip labels include the morphological species identification, followed by the sample code (see Suppl. material 2: table SS1), followed by a standard abbreviated locality for the US state (AK, CO, ID, MI, MT, OR, TN, WA, WY), Canadian province (BC, ON), or country (AUT, DEU, FIN, RUS, UK) in which the specimen was collected.

In the All *Boreus* tree (Fig. 16), four species groups were strongly supported: the first containing only sequences of *B. reductus* (Sh-aLRT = 99.9%, UFBS = 100%), the second containing *B. elegans*, *B. nivoriundus*, and *B. semenovi* (Sh-aLRT = 90.1%, UFBS = 95%), the third containing *B. nix*, *B. pilosus*, *B. gracilis*, *B. tananaensis*, *B. bomari*, and *B. brumalis* (Sh-aLRT = 99.8%, UFBS = 100%), and the fourth containing *B. timaryi*, *B. borealis*, *B. intermedius*, *B. westwoodii*, *B. hyemalis*, *B. californicus*, and *B. coloradensis* (Sh-aLRT = 99.5%, UFBS = 100%).

### Molecular species delimitation results

The AK *Boreus* dataset contained five morphologically identified *Boreus* species and one outgroup. The Alaskan *Boreus* species from this dataset were delimited into four molecular operational taxonomic units (mOTUs) by the ASAP analysis (ASAP score = 2.00, p-val (rank) = 3.999960e-05, threshold distance = 0.000648), five mOTUs by the ABGD algorithm (prior = 0.021544), four mOTUs by the mPTP analysis, six mOTUs by the GMYC analysis, and five mOTUs by the BIN algorithm (Fig. 15). The All *Boreus* dataset contained 17 morphologically identified *Boreus* species, and two outgroups. The *Boreus* sequences from this dataset were delimited into 54 mOTUs by the ASAP analysis (ASAP score = 4.00, p-val (rank) = 1.097804e-01, threshold distance = 0.000295), 20 mOTUs by the ABGD analysis (prior = 0.021544), 14 mOTUs by the mPTP analysis, 55 mOTUs by the GMYC analysis, and 34 mOTUs by the BIN algorithm (not all sequences from *B. californicus* were placed in BINs due to sequence length requirements) (Fig. 16). The average distance to the nearest species in the All *Boreus* dataset was above 2% for all species (Table 1).

**Table 1.** Intra- and interspecific Kimura 2-parameter distances for Alaskan BINs from BOLD.

Species (BIN)	Mean distance within BIN	Minimum distance within BIN	Maximum distance within BIN	Distance to nearest neighbor
*Boreus borealis* (BOLD:ACG1116)	0.066	0	0.169	6.463
*Boreus gracilis* (BOLD:AES2569)	0	0	0	2.982
*Boreus intermedius* (BOLD:AEL2631)	0.197	0	0.642	6.463
*Boreus tananaensis* (BOLD:ADR7546)	1.105	0	1.983	2.982
*Boreus timaryi* (BOLD:ACW5335)	0.395	0	1.007	7.239

*Boreus borealis* and *B. intermedius* each were delimited as monophyletic mOTUs in all analyses (Figs 15, 16). *Boreus timaryi* was also delimited as a monophyletic mOTU in all analyses except the GMYC All and GMYC AK analyses, where it was split into two mOTUs. *Boreus gracilis* and *B. tananaensis* were each delimited as monophyletic mOTUs in the ABGD AK, GMYC AK, and BIN analyses. In the ASAP All and GMYC All analyses, *B. gracilis* was delimited as a monophyletic mOTU, and *B. tananaensis* was split into three mOTUs. However, in the ASAP AK, ABGD All, mPTP AK, and mPTP All analyses, the two species were grouped into a single mOTU.

The non-Alaskan species *B. nivoriundus*, *B. elegans*, *B. semenovi*, *B. nix*, *B. pilosus*, *B. bomari*, and *B. brumalis* were delimited as a monophyletic mOTUs in at least half of the species delimitation methods, while *B. coloradensis*, *B. californicus*, *B. westwoodii*, and *B. hyemalis* were either split into multiple mOTUs or lumped into non-monophyletic mOTUs in all of the species delimitation methods.

## Discussion

### Species delimitation

The Alaskan *Boreus* species were delimited differently depending on the dataset. In the AK *Boreus* dataset analyses, only ABGD was fully concordant with the morphological species delimitations. The mPTP and ASAP species delimitations were concordant with each other, but lumped *B. gracilis* and *B. tananaensis* into one mOTU, while the GMYC delimitation was the only method that oversplit the Alaskan species and split *B. timaryi* into two mOTUs. The BIN delimitation was also concordant with the morphological species delimitation, however it used a different dataset than either the AK *Boreus* or All *Boreus* datasets, as it considers non-public sequences from the BOLD database. For the All *Boreus* dataset analyses, none of the four tested species delimitation methods were concordant with the morphological delimitation of the Alaskan species. ABGD and mPTP lumped *B. gracilis* and *B. tananaensis* into one mOTU but supported the monophyly of all other Alaskan species. Two methods oversplit the species: ASAP supported the monophyly of all species except *B. tananaensis*, which was split into three mOTUs, and GMYC split *B. tananaensis* into three mOTUs and *B. timaryi* into two mOTUs.

Within the All *Boreus* dataset, the molecular delimitation results were concordant with the morphological species in 5/5 analyses for both *B. borealis* and *B. intermedius*, 4/5 analyses for *B. timaryi* (GMYC split *B. timaryi* into two mOTUs), 3/5 analyses for *B. gracilis* (ABGD and mPTP lumped *B. gracilis* and *B. tananaensis*), and 1/5 analyses for *B. tananaensis* (ABGD and mPTP lumped *B. tananaensis* and *B. gracilis*, while ASAP and GMYC split *B. tananaensis* into three mOTUs).

**Figure 17.**
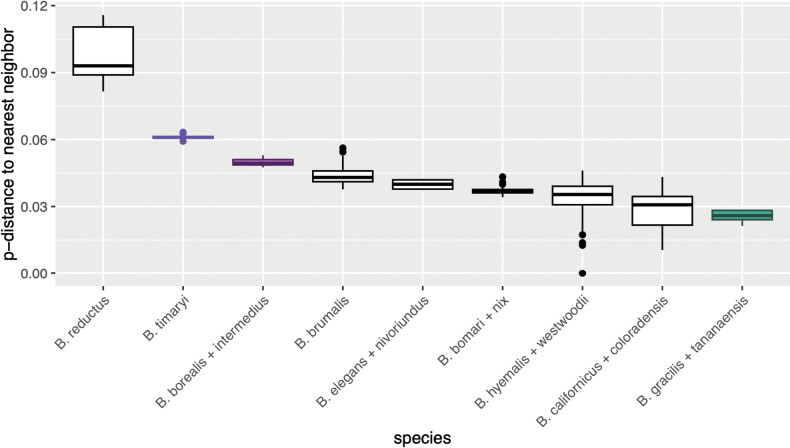
Uncorrected pairwise COI DNA barcodes distances to nearest-neighbor (NN) comparison by species, sorted from largest to smallest. Alaskan species are in color, non-Alaskan species are in greyscale. All reciprocal NNs are listed together. If NN was not reciprocal, species are listed separately.

When examining these results, it is important to consider that the *B. tananaensis* sequences have the highest interspecific diversity of any of the Alaskan species (Table 1), with a maximum interspecific p-distance of 1.98, nearly twice as high as that of the next highest (*B. timaryi*). The relatively high degree of interspecific variation in the *B. tananaensis* sequences can be partially attributed to the distance between collection localities of sequenced specimens (maximum distance = 125 km), which is nearly twice as far as the distance between collection localities for any other Alaskan *Boreus* species. Although *B. tananaensis* has not been collected from sites between Fairbanks and Quartz Lake, the species’ range may connect these two localities. Including sequences from specimens collected at intermediate localities might reduce the ‘gap’ between Fairbanks and Quartz Lake specimens, reducing the amount of oversplitting in these species delimitation methods. *Boreus tananaensis*/*gracilis* also have the lowest average distance to nearest neighbor (2.982), although the range of values between these two species overlaps with multiple non-Alaskan species pairs, including *B. hyemalis*/*westwoodii* and *B. californicus*/*coloradensis* (Fig. 17).

The results from the non-Alaskan species in the All *Boreus* dataset are similar to what was observed in Alaskan species: ASAP and GMYC tended to oversplit species when compared to the morphological delimitations, while mPTP tended towards lumping multiple species into a single mOTU. Interestingly, ABGD lumped *B. gracilis*/*B. tananaensis*, *B. westwoodii*/*B. hyemalis*, and *B. californicus*/*B. coloradensis* but oversplit *B. reductus*, and the BIN delimitation oversplit several non-Alaskan species. Overall, only five out of the 17 species included in the All *Boreus* dataset were supported by all of the tested species delimitation methods.

In some instances of species being split into multiple mOTUs, the partitions correlate with the locality where the specimens were collected. For example, in cases where *B. brumalis* was partitioned into three mOTUs, one corresponds to specimens collected in Tennessee, one to the specimen collected in Michigan, and the third to specimens collected in Ontario. In these cases, as with *B. tananaensis*, the oversplitting observed may be an artifact of geographic variation rather than true species boundaries, and evenly distributed sampling from across the species’ range might prevent such oversplitting. Likewise, some species (such as *B. gracilis* and *B. bomari*) were sampled from a single locality, and their actual genetic diversity is likely higher than is represented in our dataset.

Previous studies have documented that factors such as mutation rate, speciation rate, geneflow, population size, and sampling effect molecular species delimitation results (Dellicour and Flot 2018; Luo et al. 2018). Our results further emphasize the importance of considering not just which molecular species delimitation method to use, but also the appropriate sample size and species representation. In three out of the four species delimitation methods tested on both datasets our results varied depending on the samples used. Even in Alaskan reciprocal sister species (*B. borealis*/*intermedius* and *B. tananaensis*/*gracilis*), including non-Alaskan sequences changed how they were delimited in three out of the four molecular species delimitation methods that were tested on both datasets. The differences were also not consistent; for the ASAP and GMYC analyses, the Alaskan *Boreus* species was delimited into more mOTUs when more taxa were included in the analysis, however, for ABGD the Alaskan *Boreus* species were delimited into fewer mOTUs when more taxa were included.

### Taxonomic status of *Boreus hyemalis* and *Boreus westwoodii*

The taxonomic validity of the European *Boreus* species has been debated. The two widespread species, *B. hyemalis* (Linnaeus, 1767) and *B. westwoodii* Hagen, 1866, are found throughout the majority of Europe and are largely sympatric except in Great Britain (where *B. westwoodii* is absent) and western Russia (where *B. hyemalis* is absent) (Svensson 1972). When describing *B. westwoodii*, Hagen differentiated it from *B. hyemalis* on the basis of differences in the third tergal apophysis, differences in the shape of the hypandrium, and differences in coloration. The other currently valid European species, *Boreus lokayi* Klapálek, 1901, was described from a small number of specimens from Romania and was collected from a locality where *B. hyemalis* is also known (Svensson 1972), and is poorly known compared to the other European species.

Multiple morphological studies published have supported the validity of *B. hyemalis* and *B. westwoodii* (McLachlan 1869; Lestage 1941; Meinander 1962; Mickoleit and Mickoleit 1976), but more recent studies using both morphological and molecular data have found results inconsistent with there being two European species. In a comparative morphological study, Kreithner (2001) documented both *B. hyemalis* and *B. westwoodii* in Switzerland, but also noted the presence of three other “forms” which did not fit with either species, or which were morphologically intermediate. Kaspřák (2012) examined the validity of *B. hyemalis*, *B. westwoodii*, and *B. lokayi* from specimens collected in Czechia and Slovakia, where the ranges of the three species overlap. Kaspřák combined morphological data with three mitochondrial markers (COI, 12S, 16S) and one nuclear marker (ITS2). The results from the morphological and molecular datasets were inconsistent; specimens that were morphologically divergent shared identical sequences, while nearly morphologically identical specimens had divergent sequences. The results from the COI and ITS2 gene trees suggested *B. hyemalis* and *B. westwoodii* may each contain multiple species, while the 12S and 16S results suggested that these European species may have been historically oversplit.

Zangl et al. (2021) compared species delimitations based on COI barcode sequences to the morphology of *B. hyemalis* and *B. westwoodii* specimens. *Boreus westwoodii* was found to be paraphyletic with respect to *B. hyemalis*, and the species was divided into 2–15 mOTUs depending on the molecular delimitation method. *Boreus hyemalis* was supported as a species in all molecular delimitation methods but one, in which it was grouped with a subset of the *B. westwoodii* specimens. While the *B. hyemalis* specimens were morphologically identifiable from *B. westwoodii*, the *B. westwoodii* specimens identified by the molecular delimitation methods could not be morphologically differentiated. Zangl et al. (2021) suggest that this may indicate a cryptic species complex within the European *Boreus* species. Within their study, Zangl et al. (2021) sequenced COI barcodes for 65 *B. westwoodii* specimens and two *B. hyemalis* specimens. As shown above with Alaskan *Boreus* species, differences in sample size and representation can change species delimitation results. Strohmeier (2023) addressed the monophyly of *B. westwoodii* with the COI sequences from Zangl et al. (2021) and additional COI sequences from *B. westwoodii* specimens from southern and western Austria, as well as cuticular hydrocarbon data, and found that both genetic and chemical data support the existence of cryptic diversity in *B. westwoodii*. We re-analyzed a subset of the *B. westwoodii* from Zangl et al. (2021) in our All *Boreus* dataset (Fig. 16) and included an additional six *B. hyemalis* sequences from BOLD. This analysis included 8 *B. hyemalis* sequences from five localities in three countries and ten *B. westwoodii* sequences from nine localities in two countries. The phylogenetic results were generally concordant with Zangl et al. (2021); *B. westwoodii* and *B. hyemalis* were not found to be reciprocally monophyletic. The species delimitation results differed from those of Zangl et al. (2021) however. One species delimitation method (mPTP) grouped all *B. hyemalis* and *B. westwoodii* specimens into one mOTU, while the remaining three methods (ASAP, GMYC, BIN) split the two species into 7–11 mOTUs. Only one mOTU (found by ASAP, GMYC and BIN) consisted of both *B. hyemalis* (two sequences) and *B. westwoodii* (one sequence), potentially due to a specimen misidentification. These results are consistent with the conclusions in Zangl et al. (2021) but also emphasize the value of sequencing more *B. hyemalis* and *B. westwoodii* specimens from across the ranges of both species and the use of phylogenomic methods in addressing this question.

#### Subgeneric classification of *Boreus* species

The first effort to formally classify *Boreus* species occurred when Lestage (1940) split the genus into two genera, *Boreus* and *Euboreus*. *Boreus* was diagnosed by the presence of tergal apophyses, and *Euboreus* was diagnosed by the absence of these processes. However, Lestage’s concepts for these genera were largely ignored by the scientific community (Cooper 1951; Martynova 1954; Byers 1955; Byers 1961; Tarbinsky 1962; Caron 1967; Svensson 1972) and the two genera were eventually synonymized by Cooper (1972).

Two subgeneric-level classifications of *Boreus* have been formally suggested since Lestage (1940) (Table 2). Penny (1977) named four species groups and four species subgroups. These are classified on the basis of the presence of pronotal bristles, the fusion of the 8^th^ and 9^th^ abdominal terga and sterna, the presence of tergal apophyses on the 2^nd^ and 3^rd^ terga, and the shape of the male terminalia. Following Penny’s (1977) classification for the Alaskan *Boreus* species, *B. gracilis* and *B. tananaensis* belong in the *brumalis* subgroup of the *nivoriundus* species group (pronotal bristles present, hypandrium notched), and *B. borealis* and *B. intermedius* belong in the *californicus* species group (no pronotal bristles, 8^th^ tergum and sternum fused, tergal apophyses absent). *Boreus timaryi* is closest to the *hyemalis* species group (8^th^ tergum and sternum fused, tergal apophyses present), which otherwise contains only European species. However, the presence of short, posterior pronotal bristles in *B. timaryi* conflicts with Penny’s key as this is the first documented species outside of the *nivoriundus* species group to have pronotal bristles. However, the number and length of these bristles differ from what is seen in other species and given that pronotal bristles are present in the sister genus *Hesperoboreus*, the trait is likely ancestral to *Boreus*. *Boreus timaryi* may therefore represent an earlier branching group of the *californicus* or *hyemalis* groups that diverged before pronotal bristles were lost.

**Table 2.** Subgeneric classifications proposed for *Boreus*. The classifications by Penny (1977) consist of species groups followed by species subgroups in parentheses, if applicable. Classifications were marked as ‘unknown’ for species with insufficient information available to classify them. Species that were explicitly classified in Nikolajev (2003) or Penny (1977) are listed in **bold**; we determined the classification of the remaining species following the descriptions of the subgenera and species groups listed in Nikolajev (2003) and Penny (1977). Species that do not fit the diagnosis of the subgenera/species group they were assigned to are denoted with an asterisk. The species groups supported by the All *Boreus* dataset phylogeny (Fig. 16) are listed for species with COI sequence included in the dataset.

**Species**	**Distribution**	**Nikolajev (2003) subgenera**	**Penny (1977) species groups**	**Groups from phylogeny**
*B. hyemalis*	Palearctic	***Boreus*** (type species)	***hyemalis***	4
*B. lokayi*	Palearctic	* Boreus *	***hyemalis***	unknown
*B. westwoodii*	Palearctic	*** Boreus ***	***hyemalis***	4
*B. borealis*	Nearctic	* Boreus *	***californicus***	4
*B. californicus*	Nearctic	* Boreus *	***californicus***	4
*B. coloradensis*	Nearctic	* Boreus *	***californicus***	4
*B. intermedius*	Nearctic	* Boreus *	***californicus***	4
*B. timaryi*	Nearctic	* Boreus *	*hyemalis*	4
*B. altaicus*	Palearctic	***Euboreus***	*reductus* (unknown)	unknown
*B. beybienkoi*	Palearctic	***Euboreus***	***nivoriundus*** (*brumalis*)	unknown
*B. chadzhigireji*	Palearctic	***Euboreus***	***reductus*** (*vlasovi*)	unknown
*B. talassicola*	Palearctic	***Euboreus***	*nivoriundus* (*brumalis*)	unknown
*B. transiliensis*	Palearctic	***Euboreus***	*reductus* (unknown)	unknown
*B. vlasovi*	Palearctic	***Euboreus***	***reductus* (*vlasovi*)**	unknown
*B. brumalis*	Nearctic	*Euboreus*	***nivoriundus* (*brumalis*)**	3
*B. bomari*	Nearctic	*Euboreus*	*nivoriundus* (*brumalis*)	3
*B. gracilis*	Nearctic	*Euboreus*	*nivoriundus* (*brumalis*)	3
*B. insulanus*	Nearctic	*Euboreus*	*nivoriundus* (*brumalis*)	unknown
*B. nivoriundus*	Nearctic	***Euboreus*** (type species)*	***nivoriundus* (*nivoriundus*)**	2
*B. nix*	Nearctic	*Euboreus*	***nivoriundus* (*brumalis*)**	3
*B. pilosus*	Nearctic	*Euboreus*	***nivoriundus* (*brumalis*)**	3
*B. reductus*	Nearctic	*Euboreus*	***reductus* (*reductus*)**	1
*B. tananaensis*	Nearctic	*Euboreus*	*nivoriundus* (*brumalis*)	3
*B. jacutensis*	Palearctic	*** Fuscoboreus ***	*nivoriundus* (*nivoriundus*)	unknown
*B. jezoensis*	Palearctic	*** Fuscoboreus ***	unknown	unknown
*B. orientalis*	Palearctic	*** Fuscoboreus ***	reductus* / *nivoriundus* (*nivoriundus*)	unknown
*B. semenovi*	Palearctic	***Fuscoboreus*** (type species)	reductus* / *nivoriundus* (*nivoriundus*)	2
*B. elegans*	Nearctic	* Fuscoboreus *	***nivoriundus* (*nivoriundus*)**	2
*B. sjoestedti*	Palearctic	unknown	unknown	unknown

Nikolajev (2003) also proposed a subgeneric classification, which placed Palearctic *Boreus* species into three subgenera on the basis of the fusion of the 8^th^ tergum and sternum, and the relative width of the male forewing – subgenus *Boreus* Lestage, 1940 sensu Nikolajev 2003 (type species: *Boreus hyemalis* (Linnaeus, 1767)), subgenus *Fuscoboreus* Nikolajev, 2003 (type species: *Boreus semenovi* Pliginsky, 1930), and subgenus *Euboreus* Lestage, 1940 sensu Nikolajev 2003 (type species: *Boreus nivoriundus* Fitch, 1847). These subgenera were originally intended for classification of Palearctic *Boreus* species. Applying Nikolajev’s (2003) subgeneric classification to the Alaskan species, *B. borealis*, *B. intermedius*, and *B. timaryi* belong in the subgenus *Boreus* (8^th^ tergum and sternum fused), and *B. gracilis* and *B. tananaensis* belong in the subgenus *Euboreus* (8^th^ tergum and sternum unfused, male forewing narrow). Unfortunately, the type species for the subgenus *Euboreus* (*B. nivoriundus*) does not have the morphological diagnostic states that Nikolajev (2003) described for the subgenus. *Boreus nivoriundus* aligns more closely with Nikolajev’s (2003) description for the subgenus *Fuscoboreus* (unfused 8^th^ tergum and sternum, male forewings wide).

Our COI phylogeny supports four species groups (Fig. 16, Table 2), all of which had strong branch support. These species groups can also be diagnosed morphologically, using a mixture of the traits used by both Penny (1977) and Nikolajev (2003): group 1 – 8^th^ sternum and tergum unfused, without pronotal bristles; group 2 – 8^th^ tergum and sternum unfused, with pronotal bristles, with wide wings, large size; group 3 – 8^th^ tergum and sternum unfused, with pronotal bristles, with narrow wings, small size; group 4 – 8^th^ tergum and sternum fused. These groups do not correspond to Penny’s (1977) species groups or Nikolajev’s (2003) subgenera. Unfortunately, COI data are not available for the majority of the Palearctic species. Penny’s (1977) *brumalis* subgroup in the *nivoriundus* species group includes both Palearctic and Nearctic species, as does the subgenus *Euboreus* sensu Nikolajev (2003). However, the Palearctic species in question (*B. altaicus*, *Boreus beybienkoi* Tarbinsky, 1962, *Boreus talassicola* Nikolajev, 1998, *Boreus transiliensis* Nikolajev, 1998, *Boreus chadzhigireji* Pliginsky, 1914, and *Boreus vlasovi* Martynova, 1954) can be distinguished from Nearctic species by the short length of the female ovipositor, and one of these species (*B. chadzhigireji*) also has shortened forewings, as is seen in *B. reductus*. If included in the molecular phylogeny, it is possible that these species would group with either species groups 1 or 3, or form their own species group. The deeper splits in our phylogeny were poorly supported and thus were collapsed into a polytomy. Because of this, the relationship between these species groups is unknown.

In order to understand the relationship between these species further molecular analyses with multi-locus or genomic data and sampling of all *Boreus* species is needed. Once this relationship is better understood, it will be necessary to synonymize the subgenus *Fuscoboreus* under the older subgenus *Euboreus* (because the type species of each subgenus share the same diagnostic traits). If these subgenera are synonymized, the name *Euboreus* takes precedence as it was published first. A new subgenus or subgenera should be created to classify the majority of the species that belonged to *Euboreus* sensu Nikolajev 2003, leaving *Euboreus* with *B. elegans*, *B. nivoriundus*, and the previous members of *Fuscoboreus*. However, these changes should not be made until the relationships between *Boreus* species are better and more definitively understood.

### Future work

Much about *Boreus* and especially Alaskan *Boreus* remains unknown. Although the number of collected specimens representing Alaskan species has increased greatly over the duration of this project, these species remain poorly sampled. The specimens that have been collected are from limited geographic areas and small time frames. Because of this, our understanding of the distribution and phenology of Alaskan *Boreus* species is far from complete. Assuming the changes in coloration observed (Figs 8, 14) are consistent with the changes described in other *Boreus* species (Lestage 1941; Penny 1977), it seems likely that *B. intermedius* and *B. tananaensis* (the only two Alaskan species collected in both the fall and spring) overwinter as adults. However, neither species has been collected during the winter, so it is unclear whether they have a period of torpor or are active in the subnivean layer during the winter. The locality where *B. tananaensis* has been found has been sampled across an entire summer from April–September, and specimens were only collected in April, May, and September. *Boreus tananaensis* has been observed mating in both September and April (Fig. 9), and the only pupa collected was found in mid-September. No eggs or larvae have been found. In contrast, two of the Alaskan species, *B. timaryi* and *B. borealis* have only been collected in mid-summer (but have also received significantly less sampling effort).

Our knowledge of Alaskan *Boreus* diversity is also undoubtedly incomplete. In 2021, a *Boreus* sp. was documented on iNaturalist in Bethel, Alaska. Given the locality of this specimen, ca 450 km away from the nearest known *Boreus* population (*B. timaryi*), it likely represents another new *Boreus* species. However, efforts to collect it have been unsuccessful. Known Alaskan *Boreus* species have been found across huge latitudinal, elevational, and climatic gradients. Given how remote and sparsely sampled the majority of the state is, there are likely more undocumented species.

Understanding how Alaskan *Boreus* are related to other *Boreus* species may also help us understand the biogeographic history of the genus as a whole, which seems to be more complicated than previously hypothesized. The presence of *B. borealis* on the Pribilof Islands, which was historically a part of the Bering Land Bridge, connecting Alaska and Russia, suggests that Alaska may have acted as a stepping stone for *Boreus* dispersal either into or out of the Nearctic. The existence of *B. timaryi* connects the Palearctic species *B. hyemalis* and *B. westwoodii* to the Nearctic *californicus* species group. The group consisting of species in the subgenus *Fuscoboreus*, and *B. nivoriundus* and *B. elegans* also exhibits a Holarctic distribution, and as discussed above, *Euboreus* groups may also be Holarctic. Considering that *Boreus* is not a large genus, the relationships between the species are surprisingly unclear. Continuing to document and describe new species, both in Alaska and elsewhere, and collecting molecular data for all *Boreus* species is integral to furthering our understanding of the genus.

### Key to Alaskan *Boreus*

This key was created to aid in the identification of Alaskan *Boreus* species of both sexes. It is intended for use on alcohol preserved specimens but should work for pinned specimens as well, though some characters may not be visible. Some of the characters used in couplets may be difficult to see, due to the preservation or position of the specimen. For couplets where this may be the case, we have listed multiple diagnostic characters so specimens can be identified without dissection or clearing.

**Table:** 

1	Terminalia short, with median hood on ninth abdominal segment and two claw-like dististyles (Fig. 18A). With long, spiny, clasping forewings	**2** (male specimens)
–	Terminalia elongated into long, pointed ovipositor (Fig. 18B). Forewings reduced, pad-like	**6** (female specimens)
Male specimens
2	With bristles on the anterior and posterior margins of pronotum (Fig. 19C). Eighth tergum and sternum unfused (Fig. 20B)	**3**
–	With bristles only on the posterior margin of the pronotum (Fig. 19B), or without bristles on pronotum (Fig. 19A). Eighth tergum and sternum fused (Fig. 20A)	**4**
3	Body dark brown, with gold iridescent sheen. Hairs dense, relatively long. Hairs on the pronotum are at least half as long as pronotal bristles. Distal base of tergum 9 (below denticular area) straight, with long, skinny cleft in the middle (Fig. 21A). Epistomal suture extends laterally to intersect the margin of the maxillolabial complex, creating a triangular shape (Fig. 22A)	***B. gracilis***
–	Body brown, with blue-green iridescent sheen. Hairs dense, relatively short. Hairs on the pronotum are < 1/2 half as long as pronotal bristles. Distal base of tergum 9 (below denticular area) curved, with triangular cleft in the middle (Fig. 21B). Epistomal suture does not reach the margin of the maxillolabial complex (Fig. 22B)	***B. tananaensis* sp. nov**.
4	With transverse tergal apophyses on abdominal terga 3 and/or 4 (Fig. 23A). Bristles present only on posterior margin of pronotum, adjacent to base of wing (Fig. 19B)	***B. timaryi* sp. nov**.
–	Without tergal apophyses (Fig. 23B). Bristles absent on anterior and posterior margins of pronotum (Fig. 19A)	**5**
5	Tergum 9 median septum extends only slightly out of hood, if at all, short, does not reach denticular area (Fig. 24A, B). Hind wings with small spines on ventral surface (Fig. 25A)	***B. intermedius***
–	Tergum 9 median septum extends out of hood posteriorly, visible when viewed laterally and extends to denticular area (Fig. 24C, D). Hind wings without spines on ventral surface (Fig. 25B)	***B. borealis***
Female specimens
6	With bristles on anterior and posterior margins of pronotum (Fig. 19C). With a pair of cruciate bristles on the mesoscutellum	**7**
–	With bristles only on the posterior margin of the pronotum (Fig. 19B), or without bristles on pronotum (Fig. 19A). Without a pair of cruciate bristles on the mesoscutellum	**8**
7	Body dark brown, with gold iridescent sheen. With dense, long hairs. Hairs on the pronotum are at least half as long as pronotal bristles. Epistomal suture extends laterally to intersect the margin of the maxillolabial complex, creating a triangular shape (Fig. 22A). Distal valvulae with peg-like spines covering > 1/3 of total ovipositor length (Fig. 26A)	***B. gracilis***
–	Body brown with blue-green sheen. With dense, shorter hairs. Hairs on the pronotum are < 1/2 as long as pronotal bristles. Epistomal suture does not reach the margin of the maxillolabial complex (Fig. 22B). Distal valvulae with peg-like spines covering < or ~ 1/3 of total ovipositor length (Fig. 26B)	***B. tananaensis* sp. nov**.
8	Bristles present only on posterior margin of pronotum, with one bristle per side (Fig. 19B). Cerci < ¼ total ovipositor length	***B. timaryi* sp. nov**.
–	No bristles present on pronotum (Fig. 19A). Cerci greater than ¼ total ovipositor length	**9**
9	Distal valvulae peg-like spines tapering into fine, hairlike processes pointing posteriorly (sometimes abraded on older specimens) (Fig. 27A). Rostrum short, < 1 mm. 20–21 antennomeres. Body length < 5.5 mm from base of antennae to tip of ovipositor. Valvulae narrowed suddenly at midpoint (Fig. 28A)	***B. intermedius***
–	Distal valvulae peg-like spines do not taper into hairs (Fig. 27B). Rostrum long, > 1 mm. 19–20 antennomeres. Body length > 5.5 mm in length from base of antennae to tip of ovipositor. Valvulae taper evenly (Fig. 28B)	***B. borealis***

**Figure 18.**
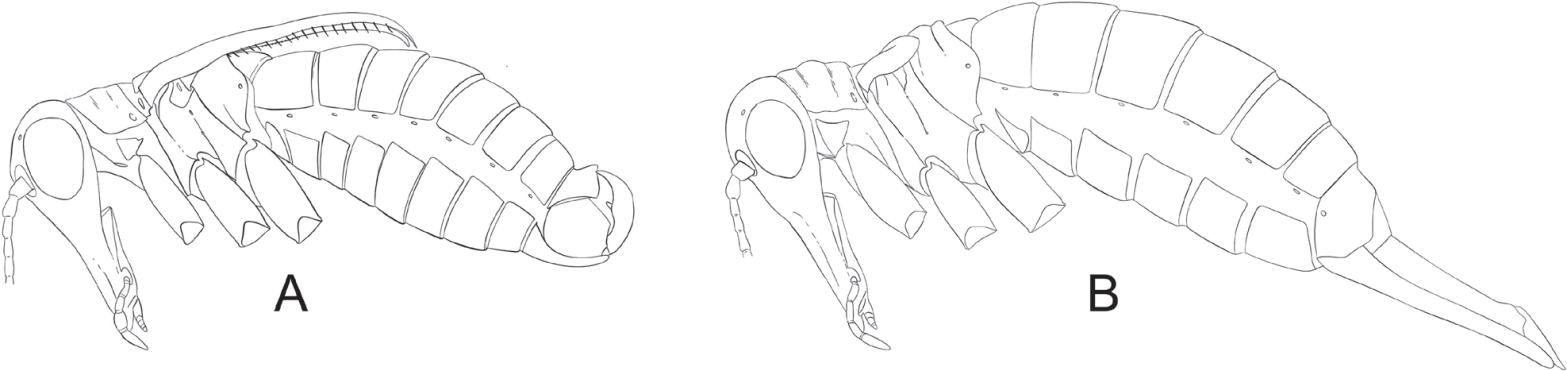
**A**. Male *Boreus* sp. and **B**. Female *Boreus* sp., viewed laterally.

**Figure 19.**
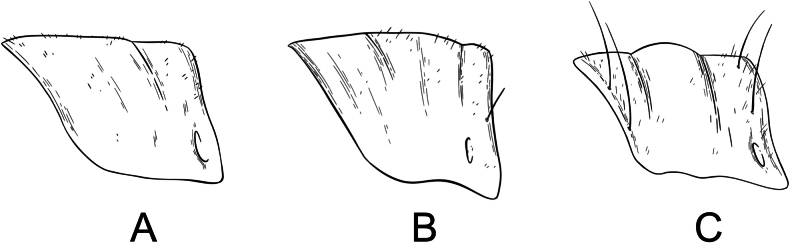
*Boreus* spp. pronota, viewed laterally, with anterior to the left. **A**. Without pronotal bristles as seen in *B. borealis*; **B**. With pronotal bristles on the posterior margin only as seen in *B. timaryi*; **C**. With pronotal bristles on the posterior and anterior margins as seen in *B. tananaensis*.

**Figure 20.**
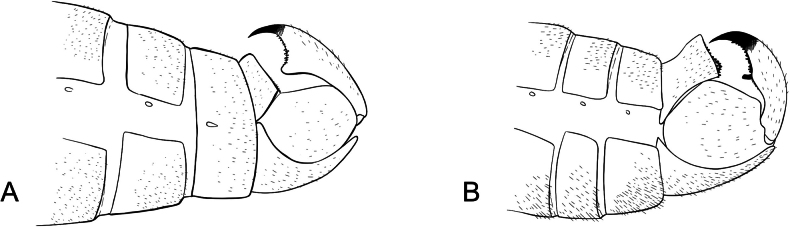
Posterior of male *Boreus* spp. abdomens viewed laterally. **A**. 8^th^ abdominal segments fused, as seen in *Boreus timaryi* sp. nov. **B**. 8^th^ abdominal segments unfused, as seen in *B. tananaensis* sp. nov.

**Figure 21.**
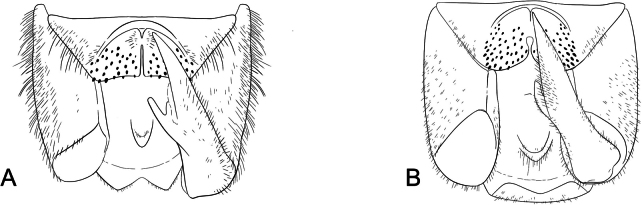
Male postabdomen viewed dorsally. **A**. *B. gracilis*; **B**. *B. tananaensis* sp. nov.

**Figure 22.**
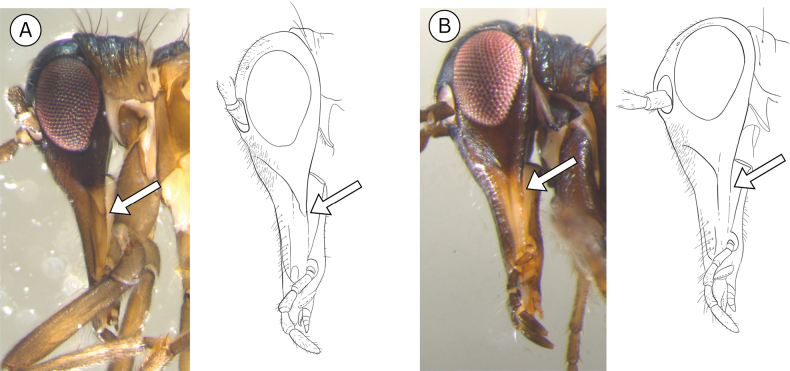
Head viewed laterally, arrows indicate lateral end of epistomal suture. **A**. *B. gracilis* (UAM:Ento:457835); **B**. *B. tananaensis* sp. nov. (UAM:Ento:504865).

**Figure 23.**
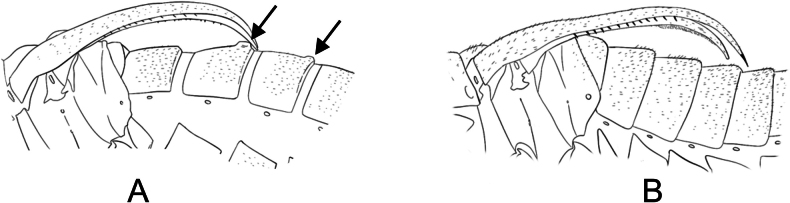
Tergal apophyses **A**. Present, as seen on *B. timaryi* (indicated by arrows); **B**. Absent, as seen on *B. borealis*.

**Figure 24.**
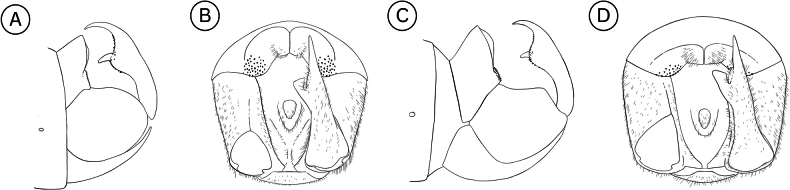
Male postabdomen of *B. intermedius* viewed: **A**. Laterally; **B**. Dorsally and of *B. borealis* viewed: **C**. Laterally; **D**. Dorsally. In **B** and **D** the left dististyle has been removed to better show the denticular area and tergal pockets.

**Figure 25.**
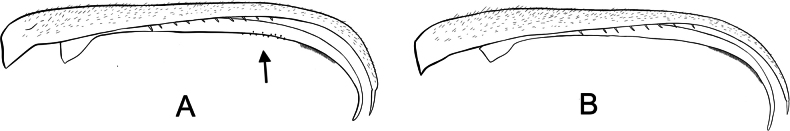
Male left fore- and hind-wings, viewed laterally. **A**. *Boreus intermedius* with arrow indicating small hind wing spines on ventral surface, and **B**. *Boreus borealis*, without hind wing spines.

**Figure 26.**
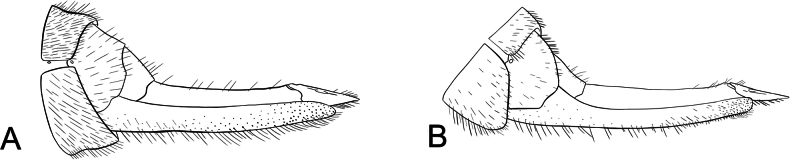
Female terminalia viewed laterally. **A**. *B. gracilis*; **B**. *B. tananaensis* sp. nov.

**Figure 27.**
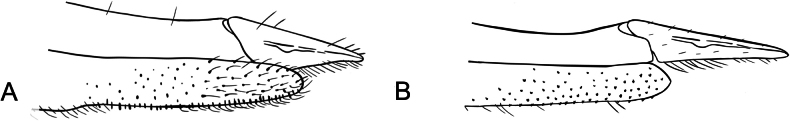
Distal end of ovipositor viewed laterally. **A**. *B. intermedius*; **B**. *B. borealis*.

**Figure 28.**
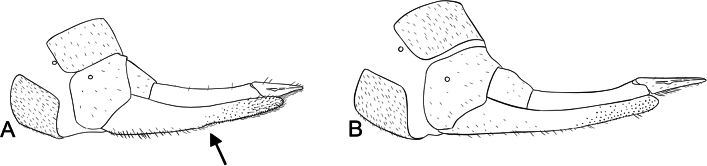
Female terminalia viewed laterally. **A**. *Boreus intermedius*, with arrow indicating where valvulae narrow; **B**. *Boreus borealis*.

### World checklist of Boreidae species (Mecoptera)

Species range data are consolidated from literature and preserved specimen records shared with the Global Biodiversity Information Facility.

#### Subfamily Boreinae Latreille, 1816

**Genus *Boreus* Latreille, 1816**

**1. *Boreus altaicus* Nikolajev, 2015**

**Distribution**. Palearctic

**Range**. KAZAKHSTAN

**Type locality**. Distvyaga Ridge, Kathon-Karagai State National Nature Park, Kazakhstan

**Type specimens**. Described from male holotype, 25 paratypes, and 84 additional specimens. The holotype was deposited at the Zoological Institute of the Russian Academy of Sciences (St. Petersburg).

**Note**. Published in Gabdullina and Nikolajev 2015.

**2. *Boreus beybienkoi* Tarbinsky, 1962**

**Distribution**. Palearctic

**Range**. KYRGYZSTAN; KAZAKHSTAN

**Type locality**. Kyrgyz Ala-Too Ridge, Kyrgyzstan

**Type specimens**. Information on type specimens not recorded in original publication. Originally deposited in Tarbinsky’s private collection. Current location unknown.

**Notes**. This species was originally published as *Boreus bey-bienkoi*.

**3. *Boreus bomari* Byers & Shaw, 1999**

**Distribution**. Nearctic

**Range**. USA: Wyoming

**Type locality**. Medicine Bow National Forest, Albany Co., Wyoming, United States

**Type specimens**. Described from holotype male, and 23 paratypes. The male holotype, allotype female, and 19 paratypes are in the collection at the University of Wyoming, Laramie.

**4. *Boreus borealis* Banks, 1923**

**Distribution**. Nearctic

**Range**. USA: Alaska, Pribilof Islands

**Type locality**. St. Paul Island, Pribilof Islands, Alaska, United States

**Type specimen**. Described from two male and two female syntypes. The male lectotype is in the MCZ. Three paralectotypes are in the USNM.

**Notes**. Banks (1923) described *B. borealis* from a series of four specimens. Subsequent publications (Carpenter 1931; Penny 1977) considered some of these specimens to be lost. Carpenter (1931) believed that Banks described the species from a pair of cotypes, the female of which had been lost. Penny (1977) believed correctly that the species had been described from a series of two males and two females, but that three of the specimens had been lost. After locating only one male specimen in the MCZ, Penny assigned this specimen as the lectotype. We confirmed the remaining three “lost” specimens that composed the original type series are in the USNM. Two of these specimens and the MCZ specimen erroneously bore paratype labels. The third USNM specimen bears a label reading “type.” In his original description, Banks specified no information about the number of type specimens. However, earlier in the publication, the fieldnotes of Alvin G. and Elsie G. Whitney are included, which indicate that four *B. borealis* were collected (Lots 127 and 159) (McAtee et al. 1923).

**5. *Boreus brumalis* Fitch, 1847**

**Distribution**. Nearctic

**Range**. USA: Illinois, Maryland, Massachusetts; Michigan, Minnesota, New Hampshire, New York, Ohio, Pennsylvania, Tennessee, Vermont, Virginia, West Virginia, Wisconsin; CANADA: Ontario, Quebec

**Type locality**. Eastern New York, United States

**Type specimens**. Information on type specimens was not recorded in Fitch (1847). The male lectotype and the male paralectotype are in the MCZ.

**6. *Boreus californicus* Packard, 1870**

= *Boreus unicolor* Hine, 1901. Synonymized in Penny (1977): “*B. unicolor* was originally described as being similar to *californicus*, but with a dark ovipositor. Dark specimens appear structurally identical to lighter specimens of *californicus*. *B. unicolor* is also similar to *coloradensis*, but the latter species does not have apical femoral spines. G. W. Byers examined the two syntypes of *unicolor* and noted spines on both hind femora. Thus, *B. unicolor* is regarded as a junior synonym of *californicus*.” Type specimens: Described from a series of two female specimens. The female lectotype and female paralectotype are in the USNM.

= *Boreus isolatus* Carpenter, 1935. Synonymized in Penny (1977): “*B. isolatus* was described as being similar to *californicus*, but with a deeply notched ninth sternum in males. While studying the holotype (and only known specimen) of *isolatus*, G. W. Byers noted that the notched ninth sternum was asymmetrical. It appeared that apical setae were adhered together with foreign material. After cleaning, the apex of the ninth sternum was found to be smoothly rounded as in *californicus*. Accordingly, *isolatus* is also placed as a junior synonym of *californicus*.” Type specimens: Described from a single male specimen. The male holotype is in the MCZ.

= *Boreus californicus fuscus* Carpenter, 1935. Synonymized in Penny (1977): “Other specimens with the more usual dark body with light appendages were used by Carpenter (1935) as basis for *B. californicus fuscus*. As noted in the section on coloration, this species generally develops the dark pigmentation in some body parts more slowly than in others. Thus, specimens with yellowish legs will have darker legs later in the season. Rusty red specimens have been found at localities having also darker and lighter specimens. For these reasons, I feel that subspecific rank for any color variants of *californicus* is unjustified.” Type specimens: Described from male holotype, 22 paratypes. The male holotype and female allotype are in the MCZ on one pin.

**Distribution**. Nearctic

**Range**. USA: Arizona, California, Idaho, Montana, Oregon, Washington; CANADA: Alberta, British Columbia, Yukon Territory

**Type locality**. Fort Bidwell, Modoc County, California, United States

**Type specimens**. The male lectotype and five paralectotypes are in the MCZ.

**Notes**. As noted by Lattin (1956) and Penny (1977), the original description (Packard 1870) and subsequent publications (Carpenter 1931; Carpenter 1935) listed the type locality for *B. californicus* as being in Siskiyou County, but Fort Bidwell is in neighboring Modoc county.

**7. *Boreus chadzhigireji* Pliginsky, 1914**

= *Boreus aktijari* Pliginsky, 1914. Synonymized by Nikolajev (2003): Nikolajev argued that the difference between the three species laid out by Pliginsky (1914) (*B. chadzhigireji*, *B. navasi*, *B. aktijari*) were all in morphological traits that vary intraspecifically in *Boreus* species (i.e., number of antennal segments, coloration). Type specimens: Described from one female, originally deposited in Pliginsky’s private collection. Current location unknown.

= *Boreus navasi* Pliginsky, 1914. Synonymized by Nikolajev (2003): see above. Type specimens: Described from three males, one female, originally deposited in Pliginsky’s private collection. Current location unknown.

**Distribution**. Palearctic

**Range**. RUSSIA: Crimea

**Type locality**. Environs of Sevastopol and Simferopol, Crimea

**Type specimen**. Described from 5 males, 17 females, originally deposited in Pliginsky’s private collection and the Zoological Institute of the Russian Academy of Sciences. Current locations unknown.

**Notes**. This species was originally published as *Boreus chadzhi-gireji*. The name is sometimes incorrectly spelled *B. hadzhigireji*.

**8. *Boreus coloradensis* Byers, 1955**

**Distribution**. Nearctic

**Range**. USA: Colorado, Montana, Utah, Washington, Wyoming

**Type locality**. Near University Camp, Boulder County, Colorado, United States

**Type specimens**. Originally described from male holotype, 32 paratypes, and eight additional specimens. Male holotype (UCMC 0000024), female allotype (UCMC 0000025), and 15 paratypes are in the University of Colorado Museum of Natural History.

**9. *Boreus elegans* Carpenter, 1935**

**Distribution**. Nearctic

**Range**. USA: Washington; CANADA: British Columbia

**Type locality**. Vancouver, British Columbia, Canada

**Type specimens**. Described from male holotype and one male paratype. The male holotype is in the CAS. The male paratype is in the MCZ.

**10. *Boreus gracilis* Carpenter, 1935**

**Distribution**. Nearctic

**Range**. USA: Eastern southcentral Alaska

**Type locality**. USA: Alaska, Between Kennicott and McCarthy

**Type specimens**. Described from female holotype and one female paratype. Female holotype originally deposited at the Burke Museum. Current location unknown. The female paratype is in the MCZ.

**Note**. *Boreus gracilis* was synonymized under *B. nix* in Penny (1977) on the basis that Carpenter had mischaracterized the shape of the male forewings of *B. gracilis*. The resurrected status of this species is discussed in detail in the results of this paper.

**11. *Boreus hyemalis* (Linnaeus, 1767)**

= *Panorpa hyemalis* Linnaeus, 1767

= *Bittacus hyemalis* (Latreille, 1805)

= *Gryllus proboscideus* Panzer, 1796. Synonymized in Latreille (1816). Type specimens: Information on type specimens was not recorded in original publication. Current location unknown.

= *Boreus gigas* Klapálek, 1901. Synonymized in Esben-Petersen (1915). Note: The name *B. gigas* first appeared in Brauer 1876, accompanied by a locality but no description and was thus a *nomen nudum*. Klapálek (1901) provided a morphological description of the two type specimens, noting that they were distinct from *B. hyemalis* in size, which might have been due to the specimens being swollen. The name became available as a result. Esben-Petersen (1915) lists *B. gigas* as a *nomen nudum* in his synonymic list of the order Mecoptera, as did Svensson (1972), and Penny (1977). Willman (1978) published a comprehensive redescription of the morphology of *B. gigas* but concluded that it showed no morphological differences from *B. hyemalis*. Type specimens: Information on type specimens was not recorded in Klapálek (1901). Current location unknown.

= *Boreus kratochvili* Mayer, 1938. Synonymized in Kreithner (2001). Type specimens: Details on type specimens were not recorded in Mayer (1938). Specimens were originally deposited in Tarbinsky’s private collection. Current location unknown.

**Distribution**. Palearctic

**Range**. AUSTRIA; BELGIUM; CZECHIA; DENMARK; ESTONIA; FINLAND; FRANCE; GERMANY; ITALY; LATVIA; NETHERLANDS; NORWAY; POLAND; ROMANIA; SWEDEN; SWITZERLAND; UNITED KINGDOM

**Type locality**. Thuringia, Germany

**Type specimens**. Information on type specimens not recorded in Linnaeus (1767). One female syntype in the collection of the Linnean Society of London, bearing the labels “hyemalis ex descr. 915”.

**Note**. This species is commonly misspelled as *B. hiemalis* in publications.

**12. *Boreus insulanus* Blades, 2002**

**Distribution**. Nearctic

**Range**. CANADA: British Columbia, Vancouver Island

**Type locality**. Summit of Camas Hill, Metchosin, Vancouver Island, BC, Canada

**Type specimens**. Described from the male holotype, ten paratypes and two additional specimens. The male holotype is in the RBCM.

**13. *Boreus intermedius* Lloyd, 1934**

**Distribution**. Nearctic

**Range**. USA: Eastern southcentral Alaska

**Type locality**. Between Kennicott and McCarthy, Alaska, USA

**Type specimens**. Described from male holotype and female allotype. The type specimens were originally deposited in the Washington State Museum, University of Washington, Seattle. However, they are no longer in the collection, and the location of these specimens has not been known since at least 1977 (Penny 1977).

**14. *Boreus jacutensis* Plutenko, 1984**

**Distribution**. Palearctic

**Range**. RUSSIA: Eastern Yakutia

**Type locality**. Aldan River, near the mouth of the Tympton River, Eastern Yakutia, Russia

**Type specimens**. Described from holotype male and two male paratypes. Holotype originally deposited at the Zoological Institute of the USSR Academy of Sciences (St. Petersburg).

**15. *Boreus jezoensis* Hori & Morimoto, 1996**

**Distribution**. Palearctic

**Range**. JAPAN

**Type locality**. Mt. Hirayama, Shirataki Village, Hokkaido, Japan

**Type specimens**. Described from male holotype, three paratypes, and three additional specimens. The male holotype is in the collection at the Entomological Laboratory, Faculty of Agriculture, Kyushu University, Japan.

**16. *Boreus lokayi* Klapálek, 1901**

**Distribution**. Palearctic

**Range**. CZECHIA; ROMANIA; SLOVAKIA

**Type locality**. Becegi Mountains, Romania

**Type specimens**. Information on type specimens not recorded in Klapálek (1901). A series of specimens identified as *B. lokayi* bearing Klapálek’s name and little other information are in the Czech National Museum, and may be the syntype series.

**Notes**. Penny (1977) stated: “This species is very similar, if not identical, to *B. hyemalis*. However, I have not seen enough variation in the second and third tergal apophyses of *B. hyemalis* to warrant synonymy at this time.” Subsequent morphological studies (Willman 1978; Kaspřák 2012; Zangl et al. 2021) have documented significant diversity in the shape of the tergal apophyses in *B. westwoodii* and *B. hyemalis*, which supports Penny’s view. However, these species names have not been formally synonymized.

**17. *Boreus nivoriundus* Fitch, 1847**

**Distribution**. Nearctic

**Range**. USA: Maine, Massachusetts, New Hampshire, New York, Tennessee, Vermont

**Type locality**. Eastern New York, United States

**Type specimens**. The male lectotype and paralectotype are in the MCZ.

**18. *Boreus nix* Carpenter, 1935**

**Distribution**. Nearctic

**Range**. USA: Montana; CANADA: British Columbia

**Type locality**. Gird’s Creek, Ravalli Co., Montana, United States

**Type specimens**. Described from male holotype, seven paratypes. The male holotype and six paratypes are in the MCZ.

**19. *Boreus orientalis* Martynova, 1954**

= *Boreus tardokijanensis* Plutenko, 1985. Synonymized in Nikolajev (2003): Nikolajev believed that *B. tardokijanensis* should be made a junior synonym of *B. orientalis* based on the illustrations in the descriptions of the two species. Type specimens: Described from a male holotype and a female paratype. Depository and current location unknown.

**Distribution**. Palearctic

**Range**. RUSSIA: Eastern Siberia

**Type locality**. Soviet harbor, Primorsky region, Russia

**Type specimens**. Described from one male, one female. Type specimens deposited at the Zoological Institute of the USSR Academy of Sciences (Leningrad).

**Notes**. Nikolajev (2003) suggested that *B. orientalis* should be made a subspecies or junior synonym of *B. semenovi* but did not have enough material from the far east species to decide conclusively.

**20. *Boreus pilosus* Carpenter, 1935**

**Distribution**. Nearctic

**Range**. USA: Montana; CANADA: Alberta, British Columbia, Yukon Territory

**Type locality**. Kaslo, British Columbia, Canada

**Type specimens**. Described from male holotype, 12 paratypes. The holotype and paratypes are in the MCZ.

**21. *Boreus reductus* Carpenter, 1933**

**Distribution**. Nearctic

**Range**. USA: Idaho, Montana, Washington; CANADA: British Columbia

**Type locality**. Kaslo, British Columbia, Canada

**Type specimens**. Described from male holotype and three paratypes. The holotype is in the Canadian National Collection.

**22. *Boreus semenovi* Pliginsky, 1930**

**Distribution**. Palearctic

**Range**. RUSSIA: Yakutia

**Type locality**. Uysky Ridge, Russia

**Type specimens**. Described from male holotype, deposited in the Zoological Institute of the Russian Academy of Sciences (Penny 1977).

**23. *Boreus sjoestedti* Navás, 1925**

**Distribution**. Palearctic

**Range**. RUSSIA: Kamchatka Peninsula

**Type locality**. Achomten Bay, Kamchatka, Russia

**Type specimens**. Described from female holotype, currently in the University of Stockholm.

**24. *Boreus talassicola* Nikolajev, 1998**

**Distribution**. Palearctic

**Range**. KAZAKHSTAN; KYRGYZSTAN

**Type locality**. Talas Alatau ridge, Kazakhstan

**Type specimens**. Described from 17 specimens. Type specimens were deposited in the Zoological Institute of the Russian Academy of Sciences.

**25. *Boreus tananaensis* Kane, sp. nov**.

**Distribution**. Nearctic

**Range**. USA: Interior Alaska

**Type locality**. Quartz Lake, Alaska, United States

**Type specimens**. Described from female holotype, 17 ♂♂ and 22 ♀♀ paratypes. The holotype and 33 paratypes have been deposited in the UAM.

**26. *Boreus timaryi* Kane, sp. nov**.

**Distribution**. Nearctic

**Range**. USA: Seward Peninsula, Alaska

**Type locality**. Bering Land Bridge National Preserve, Alaska, USA

**Type specimens**. Described from female holotype, 7 ♂♂ and 5 ♀♀ paratypes. The holotype and 9 paratypes deposited in the UAM.

**27. *Boreus transiliensis* Nikolajev, 1998**

**Distribution**. Palearctic

**Range**. KAZAKHSTAN

**Type locality**. Butakovskoye gorge, Kazakhstan

**Type specimens**. Described from male holotype and 116 other specimens. Type specimens were deposited in the Zoological Institute of the Russian Academy of Sciences.

**28. *Boreus vlasovi* Martynova, 1954**

**Distribution**. Palearctic

**Range**. TURKMENISTAN; TAJIKISTAN

**Type locality**. Unknown

**Type specimens**. Described from 153 specimens. Holotype unknown. Originally deposited at the Zoological Institute of the USSR Academy of Sciences (St. Petersburg). As of 2003, only four of the original series remain (Nikolajev 2003).

**29. *Boreus westwoodii* Hagen, 1866**

= *Boreus tarnanii* Navás, 1911; synonymized in Martynova (1954)

= *Boreus boldyrevi* Navás, 1911; synonymized in Martynova (1954)

**Distribution**. Palearctic

**Range**. AUSTRIA; BULGARIA; CZECHIA; ESTONIA; FINLAND; GERMANY; ITALY; LATVIA; NORWAY; POLAND; RUSSIA; SWEDEN; SWITZERLAND

**Type locality**. Germany, Finland, and England

**Type specimens**. No information on type specimens was included in Hagen (1866). A potential type series is present in the MCZ.

**Notes**. In the majority of publications this species has been misspelled as *B. westwoodi*.

##### Genus *Hesperoboreus* Penny, 1977

**30. *Hesperoboreus brevicaudus* (Byers, 1961)**

**Distribution**. Nearctic

**Range**. USA: Oregon, Washington

**Type locality**. Spencer Butte area, Lane County, Oregon, USA

**Type specimens**. Described from four specimens. The holotype male and allotype were deposited at Oregon State University.

**31. *Hesperoboreus notoperates* (Cooper, 1972)**

**Distribution**. Nearctic

**Range**. USA: Southern California

**Type locality**. Coldwater Canyon, Mt. San Jacinto, Riverside County, California, USA

**Type specimens**. Described from 29 specimens. The holotype male and three additional paratypes were deposited at the MCZ.

#### Subfamily Caurininae Russell, 1979

**Genus *Caurinus* Russell, 1979**

**32. *Caurinus dectes* Russell, 1979**

**Distribution**. Nearctic

**Range**. USA: Oregon, Washington

**Type locality**. Marys Peak, Benton County, Oregon, USA

**Type specimens**. Described from 75 specimens. The holotype male and one paratype were deposited in the CAS.

**33. *Caurinus tlagu* Sikes & Stockbridge, 2013**

**Distribution**. Nearctic

**Range**. USA: Southeast Alaska

**Type locality**. USA: Alaska, Prince of Wales Is. Hatchery Ck, 55.88433°N, 132.89734°W ± 26 m, 82 m elev.

**Type specimens**. Described from 37 specimens. The holotype and 19 paratypes are in the UAM.

**Notes**. When described, this species was known only from Prince of Wales Island in southeast Alaska. It has since been collected from forest habitat near Ketchikan on Revillagigedo Island (USA: Alaska, Bird Mtn trail, 55.33894°N, 131.61973°W +/- 500 m, 18 May 2013, Loren Russell, UAMObs:Ento:231332; Rainbird trail, Ketchikan, 55.35459°N, 131.6763°W +/- 100 m, 18 May 2013, Loren Russell, UAMObs:Ento:231333), and documented on iNaturalist from Sitka, Alaska on Baranof Island by Matt Goff in 2021 and 2023.

## Supplementary Material

XML Treatment for Boreus tananaensis

XML Treatment for Boreus timaryi

XML Treatment for Boreus gracilis

XML Treatment for Boreus borealis

XML Treatment for Boreus intermedius

## Additional information

### Use of AI

No use of AI was reported.

### Author ORCIDs

Taylor L. Kane https://orcid.org/0009-0007-5186-9821

Derek S. Sikes https://orcid.org/0000-0002-4336-2365

Nathan Schiff https://orcid.org/0000-0002-5595-4253

### Data availability

All of the data that support the findings of this study are available in the main text or Supplementary Information.

## Supplementary materials

Supplementary material 1 Supplementary figures S1–S3 Data type docx Explanation note Images illustrating how morphological characters were measured. https://binary.pensoft.net/file/1518870This dataset is made available under the Open Database License (http://opendatacommons.org/licenses/odbl/1.0/). The Open Database License (ODbL) is a license agreement intended to allow users to freely share, modify, and use this Dataset while maintaining this same freedom for others, provided that the original source and author(s) are credited. Taylor L. Kane

Supplementary material 2 Supplementary table SS1 Data type xls Explanation note Specimen and sequence data indicating what specimens were used for mophological comparison and molecular analyses, with collection and locality data. https://binary.pensoft.net/file/1518871This dataset is made available under the Open Database License (http://opendatacommons.org/licenses/odbl/1.0/). The Open Database License (ODbL) is a license agreement intended to allow users to freely share, modify, and use this Dataset while maintaining this same freedom for others, provided that the original source and author(s) are credited. Taylor L. Kane
