# Supplementary material for: A taxonomic review of Boreus (Mecoptera, Boreidae) with descriptions of two new Alaskan species
Source: Zookeys. 2026 Jan 23;1267:119–78. doi: 10.3897/zookeys.1267.170946 (PMC12859649; doi:10.3897/zookeys.1267.170946)
Supplement: Supplementary material 1 — Supplementary figures S1–S3 [file zookeys-1267-119_article-170946__-s001.docx]

# Supplementary Material


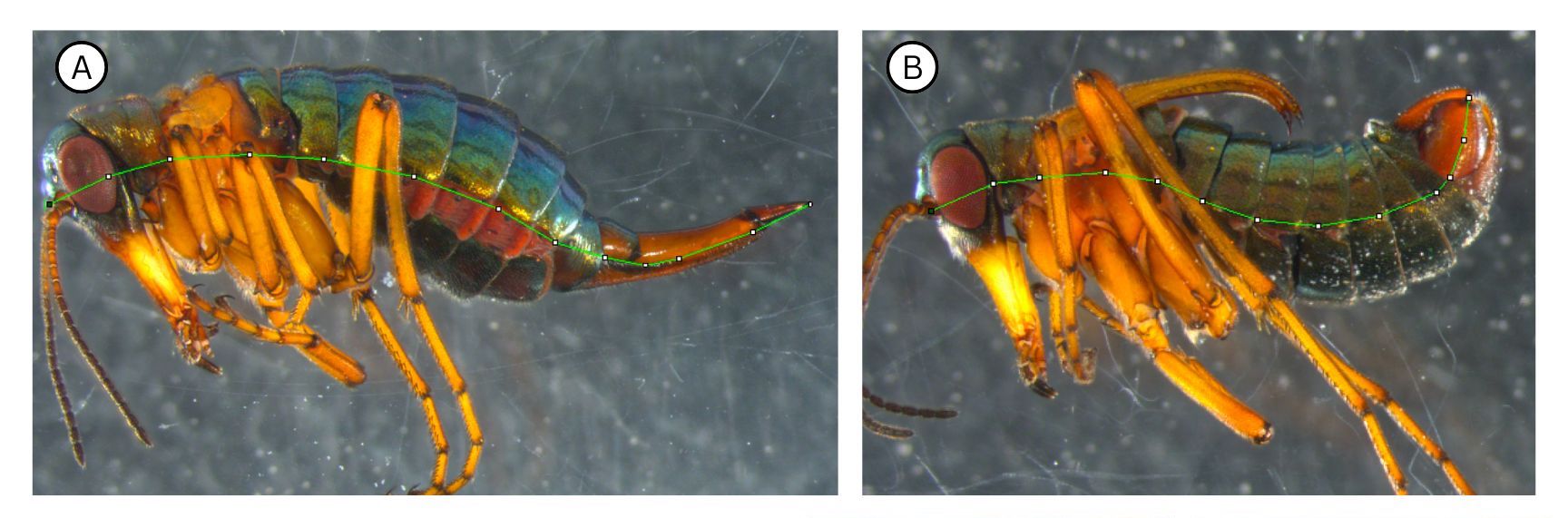


**Supplementary figure 1.** Body length was measured along the dorsal-medial line of body using a segmented line, from between antennae to **A.** the distal end of ovipositor in females and **B.** the distal end of basistyles in males.
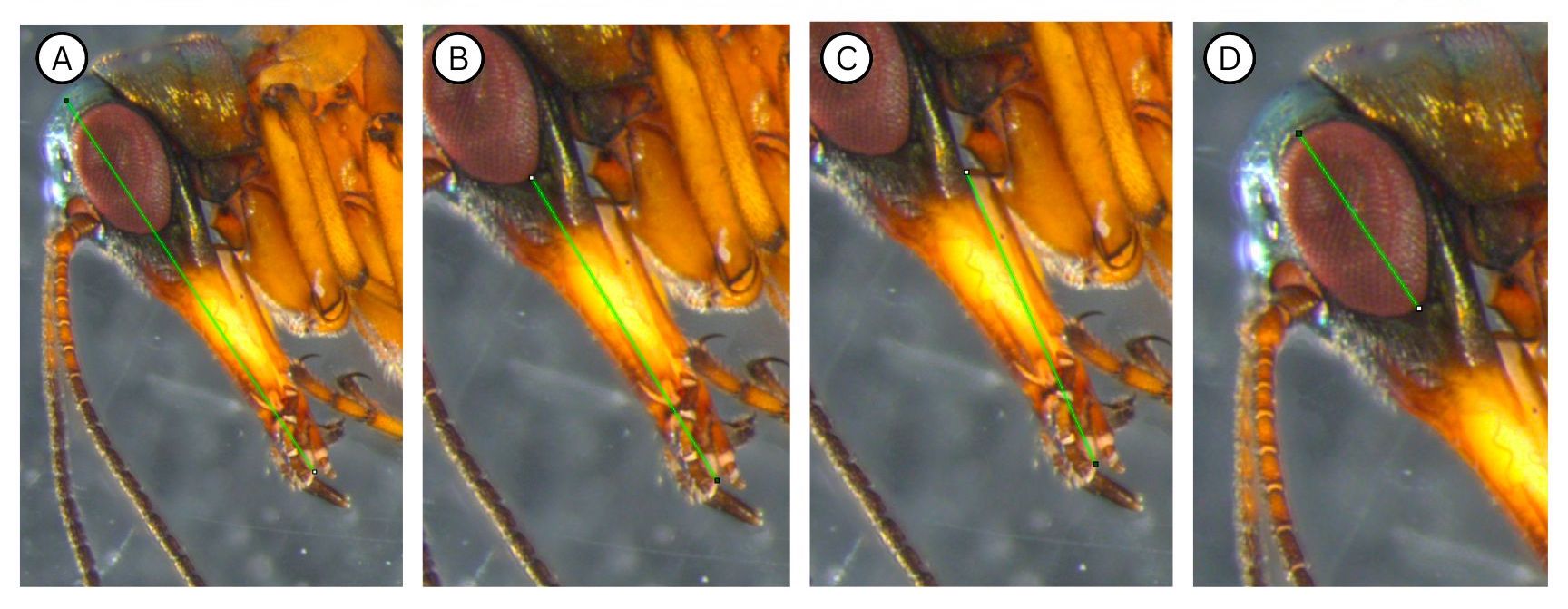


**Supplementary figure 2. A.** Head length was measured from the top of the head to the distal margin of the labrum in a straight line along the middle of the head. **B.** Rostrum length was measured from the distal margin of the eye to the distal margin of the labrum in a straight line along the midline of the head. **C.** Maxillolabial complex length was measured along from the attachment point of the cardo to the distal margin of the labrum in a straight line. **D.** Eye length was measured along the maximum diameter of the eye.


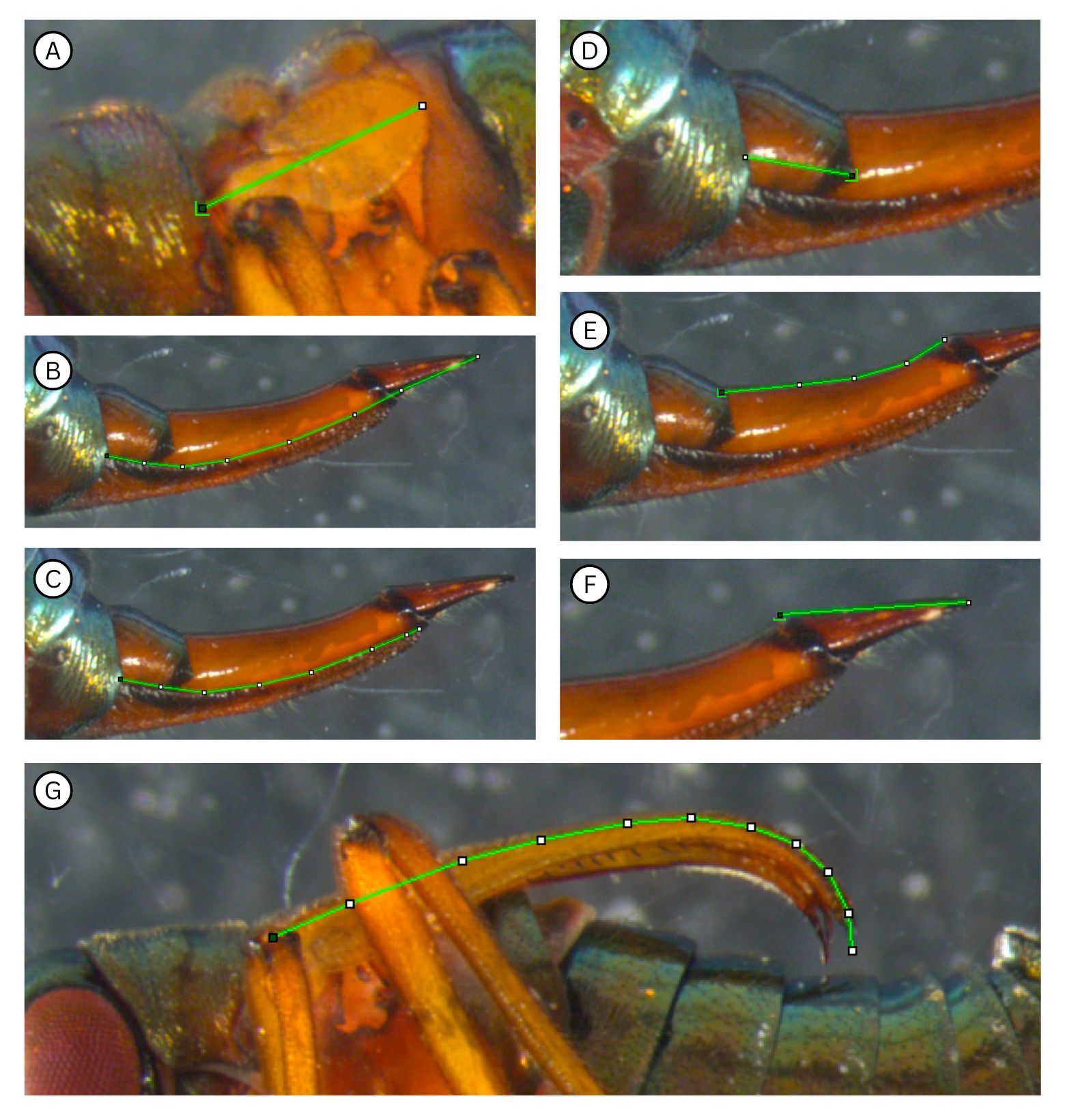


**Supplementary figure 3.** Additional character measured: **A.** Female forewing length was measured laterally from attachment point of the forewing to the most distal point in a straight line. **B.** Ovipositor length was measured laterally from the distal margin of the 8th tergum to the distal tip of the 11th tergum/cerci, measured along the midline using a segmented line. As noted by Carpenter (1935) and Penny (1977), ovipositor length can be difficult to measure as the shape of the 8th tergum varies, and the 11th segment may be deflected ventrally. Measuring the ovipositor as outlined above, using a segmented line and measuring dorsally allows for correction of these issues. Measuring the ovipositor length from the distal edge of the 8th abdominal segment, rather than the 9th abdominal segment as proposed by Penny, also allows for more meaningful comparison between the ovipositor and valvulae lengths. **C.** Valvulae length was measured laterally from the distal margin of the 8th tergum to the distal tip of the valvulae, measured along the dorsal margin using a segmented line. **D.** 9th tergum length was measured laterally from the most distal point of the 9th tergum to the distal edge of the 8th tergum, in a straight line parallel to the dorsal edge of the valvulae. **E.** 10th tergum length was measured laterally, from the distal margin of the 9th tergum to the distal margin of the 10th tergum along the dorsal edge using a segmented line. **F.** Cerci/terum 11 length was measured laterally from the proximal margin to the distal tip of segment 11 along the dorsal edge in a straight line. **G.** Male forewing length was measured laterally from the attachment point of the forewing to the most distal point in a segmented line along the dorsal margin.
